# Microfabricated Organic Electrochemical Transistors Enabled by Printing and Laser Ablation

**DOI:** 10.1021/acsami.5c16767

**Published:** 2025-11-13

**Authors:** Alan Eduardo Avila Ramirez, Jessika Jessika, Yujie Fu, Gabriel Gyllensting, Marine Batista, David Hijman, Jyoti Shakya, Yazhou Wang, Wan Yue, Renee Kroon, Jiantong Li, Mahiar Max Hamedi, Anna Herland, Erica Zeglio

**Affiliations:** † Division of Nanobiotechnology, SciLifelab, Department of Protein Science, 7655 KTH Royal Institute of Technology, Tomtebodavägen, 23a, 171 65 Solna, Sweden; ‡ School of Electrical Engineering and Computer Science, KTH Royal Institute of Technology, Electrum 229, 16440 Kista, Sweden; § Wallenberg Initiative Materials Science for Sustainability, Department of Chemistry, 7675 Stockholm University, 114 18 Stockholm, Sweden; ∥ AIMES − Center for the Advancement of Integrated Medical and Engineering Sciences at Karolinska Institutet and KTH Royal Institute of Technology, 171 77 Stockholm, Sweden; ⊥ Department of Fibre and Polymer Technology, KTH Royal Institute of Technology, Teknikringen 56, 10044 Stockholm, Sweden; # Organic Bioelectronics Laboratory, Biological and Environmental Science and Engineering (BESE) Division, 127355 King Abdullah University of Science and Technology (KAUST), 23955-6900 Thuwal, Saudi Arabia; ∇ State Key Laboratory of Optoelectronic Materials and Technologies, Key Laboratory for Polymeric Composite and Functional Materials of Ministry of Education, Guangzhou Key Laboratory of Flexible Electronic Materials and Wearable Devices, School of Materials Science and Engineering, 26469 Sun Yat-sen University, Guangzhou 510275, P. R. China; ○ Wallenberg Initiative Materials Science for Sustainability, Laboratory of Organic Electronics, Department of Science and Technology, Linköping University, Norrköping 60174, Sweden

**Keywords:** flexible electronics, organic electrochemical transistors, additive-subtractive manufacturing, sustainability, bioelectronics

## Abstract

Organic electrochemical transistors (OECTs) are key bioelectronic devices with applications in neuromorphics, sensing, and flexible electronics. OECTs made using biobased and biodegradable materials are emerging as a sustainable alternative to nondegradable plastic and metal-based electronics. Printing is the key technique used to fabricate these types of devices, enabling fabrication at room temperature and using benign solvents, such as water. However, printing techniques suffer from relatively low resolution (tens to hundreds of micrometers), far below the micrometer resolution achieved via conventional metal deposition and photolithography. Here, we present a high-throughput additive-subtractive microfabrication strategy for carbon-based flexible OECTs using biodegradable materials and room-temperature processing. Additive manufacturing of large features is achieved via extrusion printing of a graphene ink to fabricate electrode contacts on cellulose acetate (CA), which serves both as the substrate and as the insulation layer. Combined with femtosecond (fs) laser ablation, this approach enables micrometer-resolution patterning of freestanding OECTs with channel openings down to 1 μm and sheet resistance below 10 Ω/sq. By tuning laser parameters, we demonstrate both selective and simultaneous ablation strategies, enabling the fabrication of horizontal, vertical, and planar-gated OECTs, as well as complementary NOT gate inverters. Thermal degradation studies in air show that over 80% of the device mass decomposes below 360 °C, providing a low-energy route for device disposal and addressing the environmental impact of electronic waste. This approach offers a lithography-free pathway toward the rapid prototyping of high-resolution, sustainable organic electronics, combining circularity, process simplicity, and architectural versatility for next-generation bioelectronic applications.

## Graphical abstract

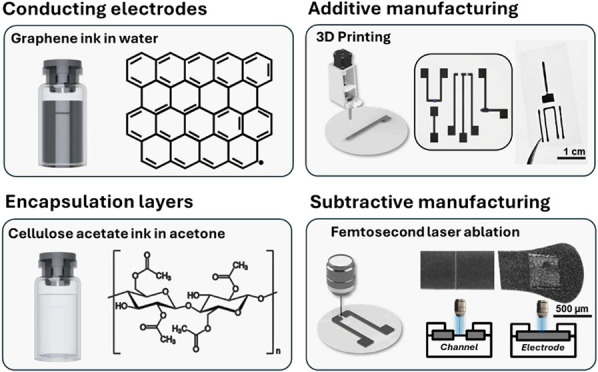

## Introduction

Organic electrochemical transistors (OECTs) leverage the unique properties of organic mixed ionic-electronic conductors (OMIECs) to combine low-voltage operation (below 1 V), high signal amplification (i.e., transconductance), and straightforward integration into mechanically flexible and conformable systems.^1^ These features make OECTs highly suitable for applications in bioelectronics, such as biosensors,^2^ neuromorphic circuits,^3^ wearable electronics,^4^ implantable therapeutics,^5^ food packaging,^6^ and plant science applications.^7^ Despite advances in the molecular engineering of OMIECs and device geometry prototyping, most high-performance OECTs are still fabricated using lithography,^8^ which remains the gold standard technique due to its high resolution (micro/nanoscale) and batch-to-batch reproducibility. However, challenges such as high costs and complex multistep processes pose barriers to fast prototyping and further scale-up, along with additional fabrication constraints due to incompatibility with biomaterials and flexible and/or degradable substrates.^8^

Additive manufacturing techniques have emerged as low-cost and scalable alternatives suitable for flexible electronics.^9^ Inkjet printing enables maskless and rapid patterning with resolutions in the 20–50 μm range for 2D structures (e.g., thin films). It has been successfully applied across various fields, such as organic photovoltaics (OPVs),^10,11^ large-scale organic light-emitting diodes (OLEDs),^12^ and quantum-dot (QD) displays.^13^ However, the development of inks for OECT fabrication is limited by the need to maintain stable jetting performance and prevent nozzle clogging,^14^ which imposes constraints on rheological properties, such as (5–20 mPa s), density (∼1000 kg m^–3^), and surface tension (20–50 mN m^–1^).^15^ The relatively high cartridge cost (>100 USD each) may also limit its use in low-cost applications, such as consumer electronic sensors.^16^ Alternatively, screen printing provides a well-established, high-throughput^17^ approach with comparable resolution (∼40 μm).^18^ This technique is user-friendly, compatible with well-established, commercially available inks, and suitable for large-scale printed electronics production. It has been explored in applications such as flexible^19^ or textile electronics.^20^ However, it requires prefabricated masks and consumes relatively large ink volumes (above 1 mL). Despite these limitations, both methods have been successfully explored for OECT prototyping^15,21,22^ and even combined in hybrid workflows for fully printed devices.^23^

However, when working with novel materials or limited ink quantities, extrusion printing has emerged as a promising prototyping alternative. Extrusion printing offers a versatile, maskless, and user-friendly approach, enabling the fabrication of structures using a wide range of materials, from conducting polymers to emerging functional inks^24^ (ink volume from 50 μL to 1 mL) to more viscous materials such as hydrogels^25^ (ink volume above 1 mL) through simple syringe-assisted (below 1 USD per unit) deposition of various inks. Depending on the needle’s inner diameter (i.e., 34 Gauge), the resolution of extrusion printing ranges from 50 to 100 μm, while micro extrusion printers can achieve resolutions as fine as 5 μm.^26^ However, rheological properties, such as low viscosity and a lack of yield stress, and speed of movement, can increase droplet or filament size beyond 200 μm.^27^ The technique has been successfully applied in OECT fabrication, including printing silver and carbon electrode contacts,^28^ and even for creating fully 3D-printed OECTs using composites like reduced graphene oxide and carbon nanotubes.^29^ Yet, additive manufacturing approaches alone still lack the resolution needed to downscale to micrometer resolution (1–10 μm), necessary for defining short channel lengths—critical to achieve higher transconductance, higher integration density, and overall highly performing devices.^30^

To address this challenge, we investigated the integration of subtractive manufacturing to complement the rapid additive pattern creation provided by additive methods with precise ablation of critical features. Femtosecond laser (fs) writing offers micrometer-scale resolution with minimal thermal damage, making it ideal for the processing of organic and carbon-based materials. This technique has been employed to pattern silver and PEDOT:PSS contacts for organic synaptic transistors and organic field effect transistors on plastic substrates.^31–33^ Moreover, we recently demonstrated that direct fs laser writing can be used to pattern insulation and active layers enabling direct, maskless patterning of OECT components with high spatial precision.^34^ However, these works used lithography and vacuum evaporation through a mask to fabricate electrode contacts and insulation layers (i.e., Parylene C).

In this work, we integrate additive and subtractive methods to overcome the limitations of each technique alone with a focus on sustainable materials and room-temperature processes. Our approach combines extrusion printing, spin-coating, and femtosecond laser ablation to produce flexible, carbon-based OECTs. Water-dispersible, 2D nanomaterials, such as graphene ink in water, offer low sheet resistance and long-term stability, making them ideal as electrode contacts for OECTs.^35,36^ At the same time, cellulose-based materials, derived from renewable sources, are abundant, biodegradable, and compatible with solvent processing, making them suitable as the substrate and encapsulation layer.^37,38^

Our fabrication protocol enables printed graphene electrodes with sheet resistance below 10 Ω sq^–1^ and critical dimensions down to 1 μm via femtosecond laser ablation. Entire device stacks remain below 10 μm in thickness, with reliable encapsulation and full delamination to produce flexible, freestanding, and thermally degradable devices. We demonstrate fabrication of horizontal and vertical OECTs, as well as in-plane gated OECTs using printed graphene as the gate. In contrast to conventional microelectrodes for OECTs, made of precious metals (i.e., gold or silver), our devices, made from organic or carbon-based materials, can degrade at lower temperatures, making them a promising alternative to improve the sustainability of organic electronics; therefore, offering a pathway for reducing e-waste.

## Materials and Methods

Graphene ink (7 wt % in water, product no. 805556), cellulose acetate (Mn ∼ 50,000, product no. 419028), and poly­(vinyl alcohol) (PVA, Mw 31,000–50,000, 98–99% hydrolyzed, product no. 363138) were purchased from Sigma-Aldrich. Poly­(3,4-ethylenedioxythiophene) doped with poly­(styrenesulfonate) (PEDOT:PSS, PH 1000) was obtained from Heraeus (Clevios). Additional chemicals—DBSA (4-dodecylbenzenesulfonic acid, product no. 44198), GOPS ((3-glycidyloxypropyl)­trimethoxysilane, product no. 440167), and ethylene glycol (EG, product no. 324558)—were also sourced from Sigma-Aldrich. The n-type semiconductor p­(C-T):PS10K 1:6 (3,7-dihydrobenzo­[1,2-b:4,5-b′]­difuran-2,6-dione) was synthesized according to previously reported literature^39^ and diluted with 10 kDaA polystyrene (1:6 monomer ratio) to improve long-term stability.^40^ The p-type semiconductor p­(g_4_2T-T) (poly­(3,3′-bis­(tetraethylene glycol methyl)-2,2′-dithiophene-thiophene)) was also prepared based on previously reported protocols.^41^ Phosphate-buffered saline (PBS 1X; pH 7.4, ∼155 mM ionic strength) was used as the electrolyte to evaluate device electrochemical performance under well-defined conditions relevant for bioelectronic applications.

### Formulation of Inks for Substrates and Insulation

A 17.5 wt % poly­(vinyl alcohol) (PVA) solution was prepared by dissolving 42.4 g of PVA in 200 mL of deionized water at 60 °C with magnetic stirring. The solution was stirred until homogeneous and left at room temperature for 24 h to achieve full clarity. For the cellulose acetate (CA) layer, we used CA with 39.7 wt % acetylation (−O–COCH_3_ groups), which is insoluble in water, leading to films with a water contact angle of ±47° measured by contact angle meter Drop Shape Analyzer-DSA 25 (KRÜSS GmbH, Germany). The CA solution was prepared by adding 4.5 g of CA in a 50 mL Falcon tube, dissolving it in 45 mL of acetone by manual agitation and allowing it to fully dissolve overnight at room temperature.

### Preparation of the Device Substrate

A 6 in. glass wafer was sequentially washed with soap and Milli-Q water and then soaked in acetone and isopropanol for 5 min for each step and then dried with air. The cleaned wafer was spin-coated with the PVA solution (8 mL, 1000 rpm, 120 s) and then placed on a hot plate at 80 °C for 20 min to dry the excess humidity. The CA solution of 8 mL was subsequently spin-coated at 500–2500 rpm for 120 s to obtain films of different thicknesses. The acetone solvent evaporated naturally at ambient temperature during the spin-coating process, forming a transparent, thin CA film.

### Fabrication of Planar OECT

All of the graphene electrodes were fabricated via direct ink writing using FELIX BIOprinter (FELIXprinters, Netherlands) using a 5 mL Omnifix syringe and nozzles (Diatom A/S) with a diameter of 0.20 mm. Electrode patterns were generated using custom-written G-code in Repetier open source software. Prior to printing, the CA substrate was air plasma-treated for 5 min using a hand-held corona surface treater (Aurora Pro Scientific). The substrate was then transferred to the bed of the printer, and the graphene ink was loaded into the syringe. After printing, the printed graphene electrodes were annealed at 130 °C for 10 min. A second layer of CA was then spin-coated on top to encapsulate the electrodes at 1500 rpm for 120 s to passivate the electrode contacts. PEDOT:PSS ink (containing PEDOT:PSS PH 1000 V/V 92.5%, GOPS V/V 1%, EG V/V 6%, and DBSA V/V 0.5% as additives) was diluted 1:4 with Milli-Q water. This formulation was then drop-cast between the femtosecond laser-patterned drain-source channels. Afterward, the PEDOT:PSS film was annealed at 120 °C for 20 min.

### Fabrication of Vertical OECTs

Fabrication of vertical OECTs for vertical devices, CA substrate, and glass wafer preparation prior to graphene printing was performed similarly to that for planar OECT fabrication. After the printing of first graphene layer, the electrodes were dried at 130 °C for 10 min. 0.2 μL of PEDOT:PSS ink (containing PEDOT:PSS PH 1000 V/V 92.5%, GOPS V/V 1%, EG V/V 6%, and DBSA V/V 0.5% as additives) was diluted 1:4 with Milli-Q water, drop-cast between source and drain electrodes, and dried at 120 °C. Afterward, a second layer of graphene was printed perpendicularly to the previous graphene layer and was dried at 130 °C for 10 min. Finally, a second layer of CA was spin-coated at 1500 rpm for 120 s to encapsulate the device prior to fs ablation on the four corners of the intersection of the *v*OECT channel avoiding ablation of graphene electrodes.

### Fabrication of Organic Complementary Inverter

OECT-based inverters used p-type polymer p­(g_4_2T-T) dissolved in chloroform (5 mg/mL) and n-type polymer p­(C-T) blended with 10 kDa polystyrene (1:6 monomer ratio) (p­(C-T):PS10K 1:6)^40^ in chloroform. Both materials were deposited as channel materials via drop-casting of 0.2 μL of solution followed by annealing at 120 °C for 20 min. An Ag/AgCl pellet was used to gate both transistors and control the input voltage (*V*_IN_), with PBS 1× as the electrolyte. The inverters were designed using channel geometry of *W* = 500 μm and *L* = 10 μm with two interconnected channels.

### Femtosecond Laser Patterning

The subtractive patterning was performed using a femtosecond laser workstation equipped with a laser source (Spirit 1040–4-SHG, Spectra-Physics, USA) and a linear motorized stage (XMS100, Newport, USA) to create the lateral opening for graphene and CA (configuration schematic is shown in Figure S17). The sample was irradiated using 4× (Plan Achromat RMS4X, Olympus, Japan), 10× (Plan Achromat RMS10×, Olympus, Japan), and 20× (Plan Achromat RMS20X, Olympus, Japan) microscope objective to focus the laser source at different beam spot size. The topmost surface interface was set by performing low-power laser irradiation, assisted by a three-dimensional ruler calibration design to define the 0 μm Z-height interface. The laser source was set to expose 520 nm laser pulse at 298 fs duration for each single pulse, where the typical working range to achieve ablation patterning occurred at 1 MHz laser pulse frequency, laser pulse energy between 300 and 1000 mW, and scan speed 10–3000 μm. Successful patterning was confirmed by upright microscope (Axiolab 5, Carl Zeiss, Germany) observation with transmitted light illumination setting to verify the patterned features.

### Electrochemical and Electrical Device Characterization

OECT and complementary amplifier devices were characterized using a Keithley 4200A-SCS parameter analyzer (Tektronix, USA), with two source measurement units (SMUs) and one pulse measurement unit (PMU). OECTs and inverter circuits were operated using PBS 1× (aqueous) solution as the electrolyte, with a silver/silver chloride (Ag/AgCl) pellet serving as the gate electrode. A Keithley 2410 source meter was connected to the parameter analyzer to supply the *V*_DD_ potential. Electrochemical characterization was performed by using a BioLogic potentiostat with an impedance module. The working electrode was a printed graphene device, the pseudoreference electrode was an Ag/AgCl pellet, and the counter electrode was a platinum electrode.

### Four-Point Probe Measurements

The sheet resistance of the thin polymer films was measured using the Ossila Four-Point Probe system with spring-loaded, rounded, gold-plated probes to ensure precise and nondestructive surface measurements. The system provides real-time current and voltage readings across the probes and incorporates both positive and negative polarity measurements to enhance the accuracy. Measurements were conducted using the integrated Ossila SMU, with results calculated using the proprietary software to account for geometrical correction factors.

### Profilometry and Feature Analysis

The thicknesses of PEDOT:PSS drop-cast films were measured using a Dektak contact profilometer and found to be 525  ±  78 nm (average of five samples). The vertical and lateral surface height profile of the patterned sample was obtained using a 3D optical profilometer based on coherence scanning interferometry (CSI) (Nexview NX2, Zygo Corporation, USA) at a 10× objective at 2× zoom to perform stitched measurement (FOV: 420 μm; vertical res: 1 nm; lateral res: 0.410 μm). The raw data were subsequently processed with a colormap to visualize surface height variations and feature recognition to calculate surface parameters using custom Python scripts. The XY coordinates and Z-values data were used to perform regional analysis and provide graphene land CA lateral opening, as well as CA depth to the graphene topmost surface (see Note S2 and Figure S4, Supporting Information).

### Raman Spectroscopy and Microscopy

Graphene inks were characterized using Raman spectroscopy on a LabRAM HR 800 Raman instrument, equipped with a 600 grating and an air-cooled double-frequency Nd:YAG laser (532 nm, 50 mW). Following femtosecond laser ablation on the device, Raman spectroscopy and spatial spectral imaging were performed on a confocal Raman imaging system alpha300 R (WITec GmbH, Germany), using a 50 × objective (Zeiss LD EC Epiplan-Neofluar Dic 50*x*/0.55) connected to a UHTS300 spectrometer. Data acquisition was conducted through a 532 nm Nd:YAG laser source with laser power of 30 mW and 0.05 s exposure time per point, with 50 × 50 raster scans over 50 μm × 50 μm to 200 μm × 200 μm designated areas for spatial imaging. Acquired spectral data was then processed and evaluated using proprietary WITec Project SIX software to conduct cosmic ray removal (filter size 7, dynamic factor 8), background subtraction (polynomial fit order 6), and TrueComponent analysis to produce the representative average spectra and intensity spatial information on each component.

### Thermogravimetric Analysis (TGA)

The devices were characterized using a TA Instruments Discovery, at a heating rate of 10 °C min^–1^ under an air flow rate of 20 mL.min^–1^. The samples were placed on a platinum HT Pan (Part number 957571.901 from TA Instruments). Reduction of noise was performed on the TGA raw signal (below 310 °C) to enhance the data clarity. For this, the OriginPro’s built-in Savitzky–Golay method was used with a polynomial of second order.

### Fourier Transform Infrared (FTIR) Spectroscopy

Several devices were placed onto an alumina crucible in a prewarmed muffle furnace at various temperatures (150, 225, 300, 450, and 600 °C) for 60 min. The samples were subsequently analyzed using a Varian 670-IR Spectrometer equipped with an attenuated total reflectance accessory using a deuterated triglycine sulfate detector (ATR-DTGS). Spectra were recorded in absorbance mode in the range 4000–390 cm^–1^ with a spectral resolution of 4 cm^–1^ and 32 scans averaged per sample.

## Results and Discussion

### Cleanroom-Free Fabrication of Encapsulated Carbon-Based Devices

Our proposed method enables the microfabrication of fully organic carbon-based OECTs by integrating both additive and subtractive manufacturing techniques. The device stack is constructed using spin-coating and extrusion printing of acetone- and water-based inks, with CA serving as both the substrate and insulating layer, and graphene as the electrode material (Figure 1a–e). To achieve microscale patterning of the channel area, we employed direct femtosecond laser ablation of the encapsulated graphene and CA layers.

**Figure 1:**
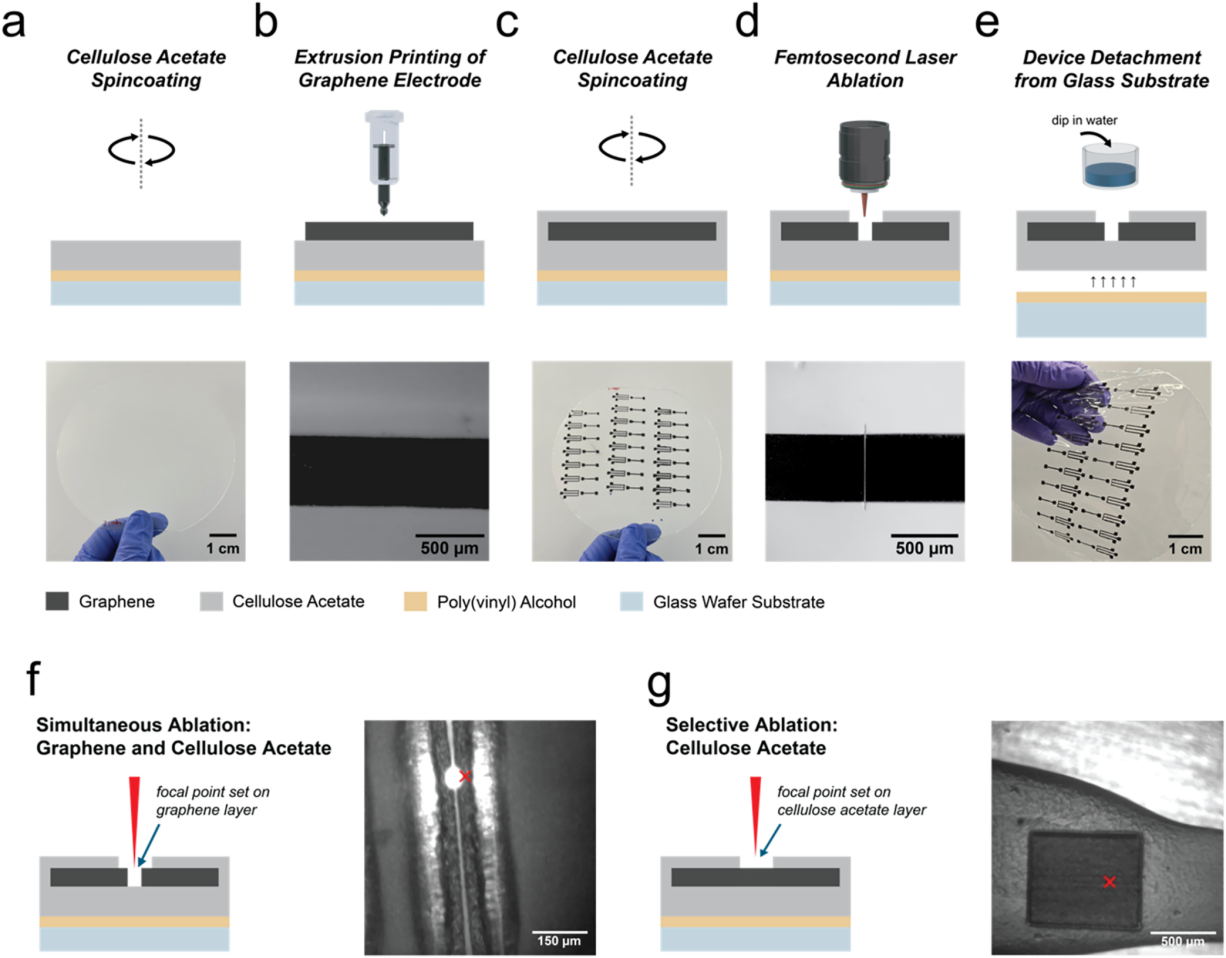
Additive-subtractive manufacturing of sustainable encapsulated electrodes for the production of OECTs. Schematic workflow of the fabrication process: (a) A sacrificial poly­(vinyl alcohol) (PVA) layer is first spin-coated onto a glass wafer, followed by spin-coating of the CA substrate. (b) Graphene ink is printed onto the CA surface to define electrode patterns. (c) After drying and annealing, a second CA layer is deposited as encapsulation. (d) Direct fs laser ablation is used to open the transistor channel or contact pad regions. (e) Devices are finally delaminated by dissolving the PVA layer in water, yielding flexible, self-standing electrode arrays. (f, g) Schematic of fs laser ablation strategies. (f) Left: simultaneous ablation of graphene and encapsulating CA layer using a 10× objective. Right: preview on the ablation process. (g) Left: selective ablation of only the CA encapsulation using a 4× objective. Right: preview on the ablation result. The *X* in red indicates the cursor inside the screen for the fs laser camera manipulation.

We chose the biobased material CA as both the substrate and passivation layer due to its transparency, film-forming properties, mechanical stability, water resistance, and degradability through chemical or enzymatic hydrolysis.^42,43^ To facilitate handling during fabrication, a 6 in. glass wafer is used as the support. A water-soluble poly­(vinyl alcohol) (PVA) layer is first spin-coated onto the wafer as a sacrificial underlayer, allowing the devices to be easily peeled off after fabrication through simple dissolution of PVA in water (see the Experimental Section for details).

The CA substrate is prepared by spin-coating from an acetone solution to create a CA film, as shown in Figure 1a. Film thickness is determined by adjusting the spin speed, with typical values ranging from 500 to 2500 rpm, yielding thicknesses ranging from 9.82 ± 0.36 μm to 3.46 ± 0.30 μm, respectively (Figure S1a). These values are comparable to typical thicknesses of OECT substrates, such as Parylene C or polyimide,^44^ but with the added benefits of avoiding energy-intensive chemical vapor deposition. For device fabrication, we selected a CA thickness of ∼5 μm (spin speed of 1500 rpm) as the substrate, providing sufficient mechanical stability following delamination from the glass wafer (Figure S1b).

Prior to printing the electrode contacts, the CA film is surface treated with a hand-held corona surface treater for 5 min to enhance graphene adhesion (see the Experimental Section). We then used 7 wt % graphene ink in water to 3D print 20 electrode patterns supporting three different architectures: standard horizontal OECTs with an Ag/AgCl gate, planar OECTs with a printed graphene gate, and vertical OECTs. A single pass through a 200 μm diameter nozzle results in electrode lines with a final line width of around 500 μm (Figure 1b), consistent with the lateral spreading of the printed pattern during drying at room temperature (1 min) prior to thermal annealing. Previous reports have shown that annealing improves the conductivity and adhesion of printed graphene to glass substrates.^45^ We investigated annealing temperatures ranging from 100 to 150 °C for 10 min, which is within the range of the glass transition temperature of CA, but below the melting temperature. Four-point probe measurements confirm a stable sheet resistance of 5.9 ± 1.1 Ω sq^–1^ across this temperature range (Figure S2a). Raman spectra reveal a red shift in the D and G bands up to 100 °C, similar to what is reported in the literature (Figures S2b and S3), consistent with the multilayer nature of graphene.^46^ No further changes in the Raman spectra were observed beyond that temperature, confirming that the annealing step does not negatively impact the structure of printed graphene films. Based on these results, we chose 130 °C as the optimal annealing temperature to ensure effective water removal without damaging the underlying CA substrate.

Following electrode printing and thermal annealing, we performed a second spin-coating step to deposit the insulation layer, resulting in a uniform, self-standing, wafer-sized multielectrode structure (Figure 1c, see the Experimental Section). A subsequent subtractive patterning step using femtosecond laser ablation was then employed to define the channel area with micrometer resolution, surpassing the resolution limitations of the printed features (Figure 1d). Successful channel opening was confirmed via optical microscopy in transmission mode (Figure 2a). The final delamination step from the glass wafer yielded flexible, self-standing devices with fully encapsulated graphene electrodes (Figure 1e).

**Figure 2:**
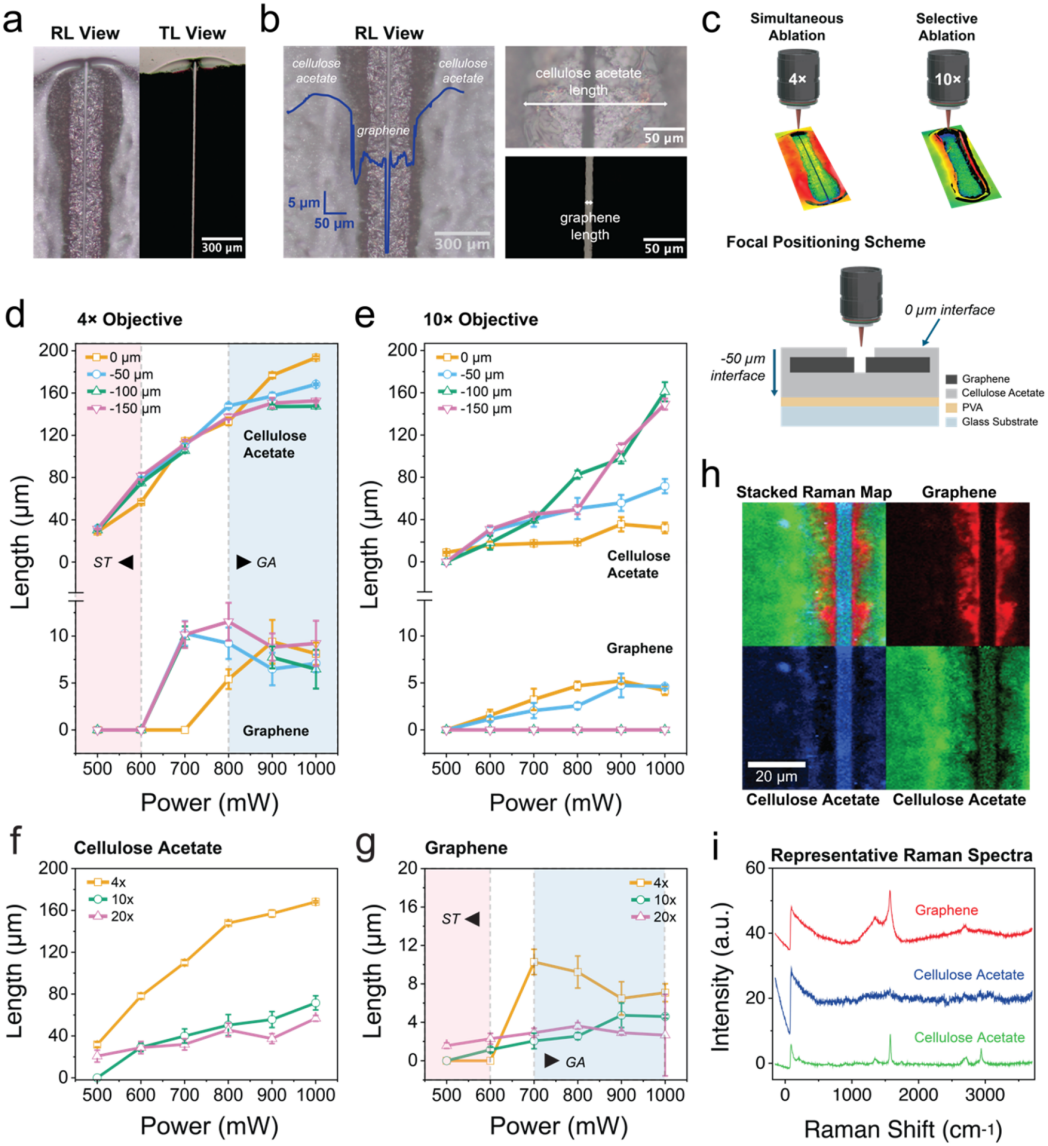
Femtosecond laser ablation parameters for the single-pass simultaneous patterning of graphene and CA. (a) Successful patterning was verified by upright optical microscopy, with corresponding reflecting (RL) and transmitting (TL) light images showing the opened channel. (b) On the left, microscope image (RL) and Z-height profile obtained from 3D optical profilometry (in blue) showing the surface morphology of CA and underlying graphene across the ablated region. On the right, magnified reflecting (top right) and transmitting (bottom right) images showing the channel opening following a single pass laser ablation, with the exposed graphene and CA lengths quantified in the next plots. (c) On the top, profilometry scans for simultaneous or selective ablation, and schematic illustration of the focal positioning strategy. Ablation depth is tuned by adjusting the Z-height from 0 to −150 μm. On the bottom, schematic illustration of the fs laser ablation process using a 4× objective for simultaneous removal of CA layer and underlying graphene. (d, e) Lateral opening dimensions for (d) CA and (e) graphene at varying Z-heights (0 to −150 μm), confirming depth-dependent ablation behavior. The graphene ablation profile marked as GA indicates the region where both CA encapsulation and graphene are ablated, while ST is an indication for subthreshold region, where only the CA encapsulation layer is ablated, but not graphene. (f, g) Quantified lateral opening lengths in (f) CA and (g) graphene as a function of laser power at different objective magnifications. (h) Confocal Raman microscopy of laser-ablated regions following simultaneous ablation with 10× objective, showing spatial separation between graphene (red) and CA (green/blue). (i) Representative Raman spectra extracted from confocal Raman data in (h), confirming material identity and quality: graphene (D, G, 2D peaks) and characteristic CA signatures from the encapsulating and substrate layers.

To demonstrate the flexibility of our fabrication method, we implemented two device architectures: conventional horizontal OECT (*h*OECT) and emerging vertical OECT (*v*OECT). While *h*OECTs are widely used and well established, *v*OECTs offer key advantages, such as reduced channel dimensions with shorter effective channel lengths and the possibility to increase device density via vertical stacking.^47,48^ Both architectures were fabricated with diluted PEDOT:PSS as the channel material to realize the depletion-mode OECTs. Schematics of the respective fabrication steps are shown in Figure 1f,g.

### Simultaneous Graphene–Cellulose Acetate (CA) Ablation for Horizontal OECTs

We systematically investigated how laser processing parameters influence the lateral opening profiles of both CA and graphene layers during a single, simultaneous femtosecond laser ablation step (analyzed via 3D optical profilometry, Figure S4). Parameters evaluated include laser pulse power, focal offset (Z-height), objective magnification, and scanning speed (Figures 2a–g and S4–S8, Supporting Information).

The focal point position (Z-height) plays a critical role in determining which layer is effectively ablated (Figure 2d,e). We restricted the focal point variation to a Z-height range down to −150 μm as further defocusing no longer influences the topmost surface. At 4× magnification, we observed simultaneous ablation of both graphene and CA encapsulation layer across a broad Z-range (0 to −150 μm, Figure 2d). This is due to the larger depth of field of the 4× objective, distributing the laser fluence across a broader volume. In contrast, at 10× magnification, we observe graphene ablation only within a limited focal offset range, down to approximately −50 μm from the CA surface, whereas CA exhibits a broader ablation tolerance down to −150 μm (Figure 2e). Beyond −100 μm, laser energy becomes insufficient to ablate graphene regardless of the power level, indicating the existence of a focal threshold specific to graphene. At 20× magnification, graphene ablation occurs at a very narrow focal range (Z-height at −50 μm) and became highly sensitive to alignment, making it more complicated to use in a reliable manner (data not shown).

Increasing the laser power from 500 to 1000 mW at a uniform focal point of −50 μm results in a wider lateral opening in both materials (Figure 2f,g). After a single pass of simultaneous laser ablation, CA exhibits a consistent expansion in opening length from around 30 μm to over −150 μm, while graphene opening lengths range from around 1 to 12 μm depending on the magnification of the objective. Ablation performed with a 4× objective produces greater variability in graphene opening length, whereas 10 and 20× objectives yield more gradual and controlled increase (Figure 2f,g). These results demonstrate that the 10 × objective, with its narrower depth of field than the 4× objective, exhibits a more selective ablation of either CA encapsulation layer or both CA and graphene layers.

Based on these data, we selected the 10× objective for all subsequent simultaneous ablation processes, as it provides an optimal balance between spatial resolution, fabrication robustness, and ease of operation. The lateral opening profile across all three objectives confirms that the 20× objective offers only minimal resolution improvements over the 10× objective while significantly complicating focus adjustment (Figure 2g). The 10× magnification configuration thus offers improved spatial resolution and ablation control compared to 4×, without the alignment challenges associated with 20× magnification.

Scanning speed and number of passes were further investigated to assess the robustness of the simultaneous ablation process. While the lateral opening dimensions remain relatively consistent across different conditions (Figure S5, Supporting Information), slower scanning speeds result in higher local laser fluence, leading to pronounced vertical bulging along the sidewalls of the surrounding CA (Figure S8, Supporting Information). This bulging is attributed to excessive heat accumulation, which causes localized melting and flow-induced material deformation at the edges of the ablated region. As the scanning speed increases, the extent of bulging decreases, indicating that shorter dwell times reduce thermal buildup and allow for more efficient heat dissipation. Thereby, we recommend a scanning speed of 1000–3000 μm s^–1^ as an optimal trade-off between the minimal thermal bulging effect and process throughput.

We observed similar fabrication effects when comparing single-pass and double-pass ablation at varying scan speeds (Figures S6–S7, Supporting Information). The additional energy delivered during the second pass has minimal impact on the lateral opening of CA and graphene, as the ablation threshold had already been reached in the initial pass. Since the second pass does not significantly increase the lateral ablation dimensions, it proves beneficial in reducing residual CA on the graphene surface. Thus, performing double passes is advisable to help improve surface cleanliness without affecting the lateral opening. Notably, we found that the presence of graphene enhances the efficiency of CA ablation. The different ablation responses of graphene and CA to single-pass laser exposure are attributed to the distinct ablation mechanisms of the two materials: nonlinear optical absorption in graphene versus thermally driven ablation in CA (see Note S1 and Figure S9 Supporting Information).

To further complement the information from static microscopy and profilometry analyses, we include Supporting Videos S1, S2, S3, and S4 that visualize the femtosecond laser ablation process in real time. These videos demonstrate both simultaneous ablation of graphene and CA (Videos S1, S2, S3, and S4) and selective ablation of the CA layer alone (Video S4). They highlight dynamic factors such as laser pass number, subthreshold optimization, and visual cues such as channel darkening and illumination angle that assist in verifying successful ablation (see Supporting Information, Video Captions for detailed annotations).

To validate channel formation and material selectivity, we performed confocal Raman microscopy across the patterned regions. The spatially resolved Raman intensity map (Figure 2h) shows three distinct regions: (i) graphene electrodes on both sides of the channel (red); (ii) the cellulose acetate substrate exposed within the channel opening (green); and (iii) the cellulose acetate encapsulation layer (blue), all confirming the successful formation of the channel. The representative Raman spectra (Figure 2i) extracted from different spatial localizations display the characteristic signatures of graphene (D, G, and 2D peaks) and confirm that the cellulose acetate substrate retains a molecular signature similar to that of the unaffected encapsulation layer. The Raman mapping validates that the fabrication process preserves the integrity of the materials across the different layers, including the insulation layer above the graphene contacts and the substrate beneath the channel. These results support further use of simultaneous graphene-CA ablation for channel formation in horizontal OECTs.

Although fully additive manufactured 3D-printed OECTs have been explored and successfully demonstrated, this approach remains limited by relatively low resolution, typically ranging between 120 and 150 μm.^29^ Here is where subtractive manufacturing can contribute to further improving the resolution. Based on the results of laser processing parameters and their effect on channel opening profiles, we selected a laser power of 500 mW at −50 μm interface with a 10× objective as the optimal conditions for patterning of horizontal OECT (*h*OECT) channels (Figure 3a). The whole fabrication process is outlined in Figure S10. The resulting *h*OECT channels have a width of 500 μm and a minimum channel length of 1.25 μm (Figure 3b). Optimization of micrometer-scale patterning can be observed in Figure S11.

**Figure 3:**
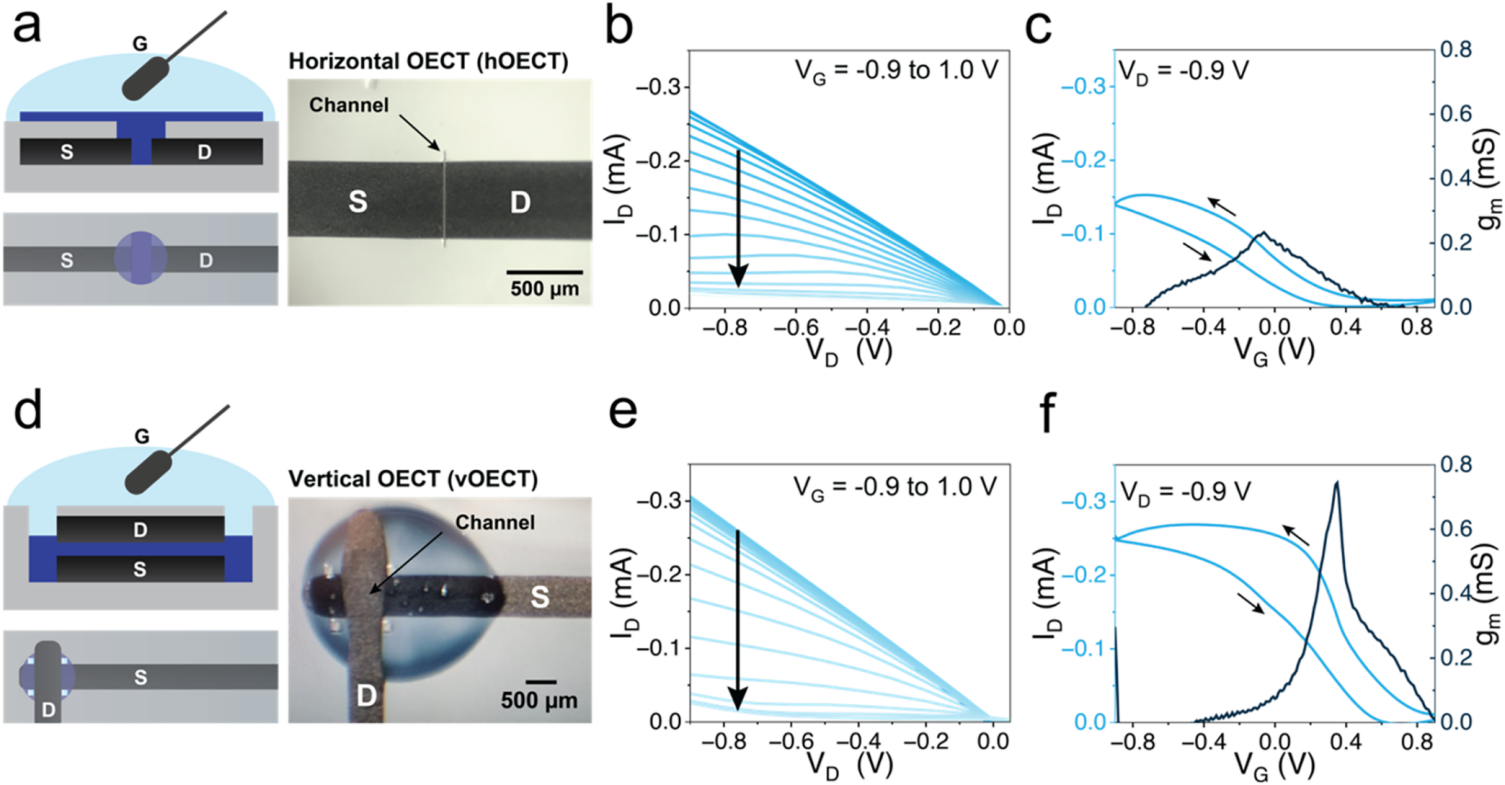
Fabrication approach and characterization of different OECT architectures. (a) Device schematic represented by the (top) cross-sectional view and (bottom) top view, with a microscope image of an ablated drain-source opening used for horizontal OECT (*h*OECT) architecture. (b) Representative output characteristics of *h*OECT device. (c) Representative transfer characteristics and transconductance of *h*OECT (*W =* 500 μm, *L =* 1 μm). (d) Vertical OECT (*v*OECT) architecture, with (top) cross-sectional view and (bottom) top view, also the microscope image of a fully fabricated *v*OECT. (e) Representative output characteristics of *v*OECT, with ablation performed after the encapsulation of electrodes and the PEDOT:PSS channel. (f) Representative transfer characteristics and transconductance of *v*OECTs (*W =* 500 μm, *L=* drop-cast PEDOT:PSS, *and d ≈* 525 μm). These measurements were performed using PBS 1× electrolyte and a Ag/AgCl pellet as the gate electrode.

Following laser patterning, the PEDOT:PSS dispersion was drop-cast onto the channel area (see the Experimental Section for details). Output characteristics show that PEDOT:PSS *h*OECTs operate in hybrid mode for negative drain voltages and gate voltages (*V*_G_) between −0.9 and 1 V, as shown in Figure 3b. These results are consistent with previous PEDOT:PSS-based OECTs operating in depletion or hybrid mode depending on the channel width-to-length ratio and thickness.^49^ Transfer characteristics measured at *V*_D_ = −0.9 V reveal a threshold voltage (*V*_TH_) of 0.8 ± 0.1 V and a maximum transconductance of 0.3 ± 0.1 mS at *V*_G_ = 0.1 ± 0.1 V (Figure 3c and representative gate currents in Figure S12). These results confirm that this laser patterning method can be successfully employed to fabricate *h*OECTs.

### Selective CA Ablation for Vertical OECTs

Vertically stacked OECT configurations, where the channel is sandwiched between the source and drain electrodes, have been introduced to reduce the channel length and enhance transistor density for circuit-level applications.^50^ An insulation layer is typically used to prevent direct exposure of the electrode contacts to the electrolyte during electrical characterization.^51^ Cicoria et al. demonstrated one of the first examples of printed vertical OECT using a printed circuit board (PCB) printer.^51^ However, incorporating the insulation layer introduces an additional challenge: creating defined pathways that allow the electrolyte to access the channel, which is essential for device operation.

Here, we employed a fabrication process modified from that used for *h*OECTs to accommodate the vertical architecture, where the conducting polymer resides between the source and drain electrodes (Figure 3d). The fabrication process begins with printing and annealing the source electrode on the CA substrate, as previously described. PEDOT:PSS is drop-cast directly onto the source contact and annealed (see the Experimental Section, Figure S10b). A second graphene layer is then printed perpendicular to the first electrode to form the drain electrode. The entire device stack is subsequently encapsulated with a spin-coated CA layer for complete electrode insulation. To enable contact between the electrolyte and the channel material, we used the femtosecond laser to ablate an opening in the CA just outside each of the four corners of the channel area of 125 μm versus 125 μm. These four openings give short diffusion distances for efficient electrochemical dope the *v*OECT channel.

This exposed the underlying PEDOT:PSS channel to the electrolyte without directly patterning the graphene, as illustrated in Figure 3d. The channel length was determined by the thickness of the drop-cast PEDOT:PSS film (Dektak, Experimental Section).

Output characteristics indicate that the *v*OECT operates at similar drain and gate voltage ranges as the *h*OECT, but with higher maximum drain currents (Figure 3e), confirming that the electrolyte can indeed reach the PEDOT:PSS channel through the ablated areas. Transfer curves reveal that *v*OECTs exhibit a sharper ON/OFF transition at a more positive regime, with a threshold voltage shift to a more positive potential of *V*_TH_ = 0.9 ± 0.2 V (Figures 3f and S12). These changes result in an increased maximum transconductance *g*_m_ = 0.7 ± 0.2 mS at *V*_G_ = 0.6 ± 0.2 V, which is consistent with shorter channel lengths compared to *h*OECTs^52^ (525 nm with respect to 1 μm for *h*OECTs, see Experimental Section). Additionally, gate currents for *h*OECT and *v*OECT are reported in .

Although our printed graphene electrodes show low sheet resistance (below 10 Ω·sq^–1^), they remain more resistive than metallic contacts, such as inkjet printer gold nanoparticles (below 2.5 Ω·sq^–1^),^53^ or chemically vapor deposited gold (2.44 μΩ·sq^–1^).^54^ This higher resistance might limit transistor performance.^55^ Moreover, drop-casting the mixed ionic/electronic conductor is expected to result in lower mobility per volumetric capacitance (μC*) with respect to other casting techniques, such as spin-coating.^56^ Future work should focus on optimizing OMIEC deposition, using higher performing p/n OMIECs, as well as exploring improvements on the treatment of graphene electrodes to minimize contact resistance.^57^

### Graphene-Based Gate Electrodes and Organic Electrochemical Inverters

Developing a method to expose graphene electrodes without compromising their integrity is essential to fabricating planar and cyclic oxide (OECT) structures with printed gates. Building upon the method of simultaneous laser ablation of graphene and CA, we established a selective ablation strategy that effectively removes only CA encapsulation while preserving the underlying graphene electrodes. We adjusted the Z-height to shift the focal point from the graphene surface to the air-encapsulation layer interface. The laser power was reduced to 500 mW (using a 4× objective), below the graphene ablation threshold but sufficient to ablate CA (Figure 2f,g). The process begins with an initial slow scan at 100 μm/s, followed by a fast pass at 3000 μm/s to remove the residual polymer. We used the method both to open planar gates and to open contact pads (Figure S13).

We performed electrochemical analysis using a three-electrode setup on 3 mm × 3 mm (9 mm^2^) patterned graphene electrodes (Figure S14) to assess successful electrode opening and stability in PBS 1× (aq.). The open-circuit potential (OCP) remained stable near 0 V for 1 h, indicating reliable operation without delamination.^58^ Cyclic voltammograms show quasirectangular shapes with minimal redox peaks, confirming the electric double-layer capacitance. Scan rate–dependent CVs (20–500 mV/s, 10 cycles) remain symmetric and reversible, exhibiting a proportional current–scan rate relationship. This behavior indicates stable, purely capacitive characteristics with minimal polarization.^59^ Impedance spectroscopy reveals a relatively low impedance (10^2^–10^3^ Ω) from 1 Hz to 100 kHz at 0.0 V, indicating rapid charge transfer and minimal resistive losses. Based on these results, we tested in-plane graphene electrodes as planar gates for *h*OECTs.

Output and transfer characteristics show that the devices operate in a hybrid mode, similarly to *h*OECTs with identical channel dimensions (*W* = 500 μm and *L* = 1 μm) with Ag/AgCl as the gate. The planar OECTs show a similar *V*_th_ = 0.8 ± 0.3 V, along with increased hysteresis between the forward and reverse sweep, and a decrease in transconductance peak of *g*_m_ = 0.2 ± 0.1 mS at higher *V*_G_ = 0.2 ± 0.1 V, compared with the *h*OECT gate with Ag/AgCl. Such a decrease in performance with respect to devices having an Ag/AgCl gate is attributed to the polarizable nature of the graphene gate in contrast to the nonpolarizable behavior of Ag/AgCl.

The depletion-mode operation of conducting polymer PEDOT:PSS limits full current switching, making these devices less ideal for digital logic circuits where a clear OFF state is required. To further demonstrate the versatility of our platform for integrating p/n organic semiconductors and circuit configurations, we developed complementary inverter circuits by integrating p-type and n-type OECTs (Figure 4d). The p-type OECT used p­(g_4_2T-T) as the channel material, while the n-type counterpart was based on p­(C-T):polystyrene 10 kDa (1:6) blend, selected for its improved operational stability with respect to pristine p­(C-T) (Figure 4f–g).^60^ Both materials were deposited by drop-casting on laser-patterned channels with *W* = 500 μm and *L* = 10 μm.

**Figure 4:**
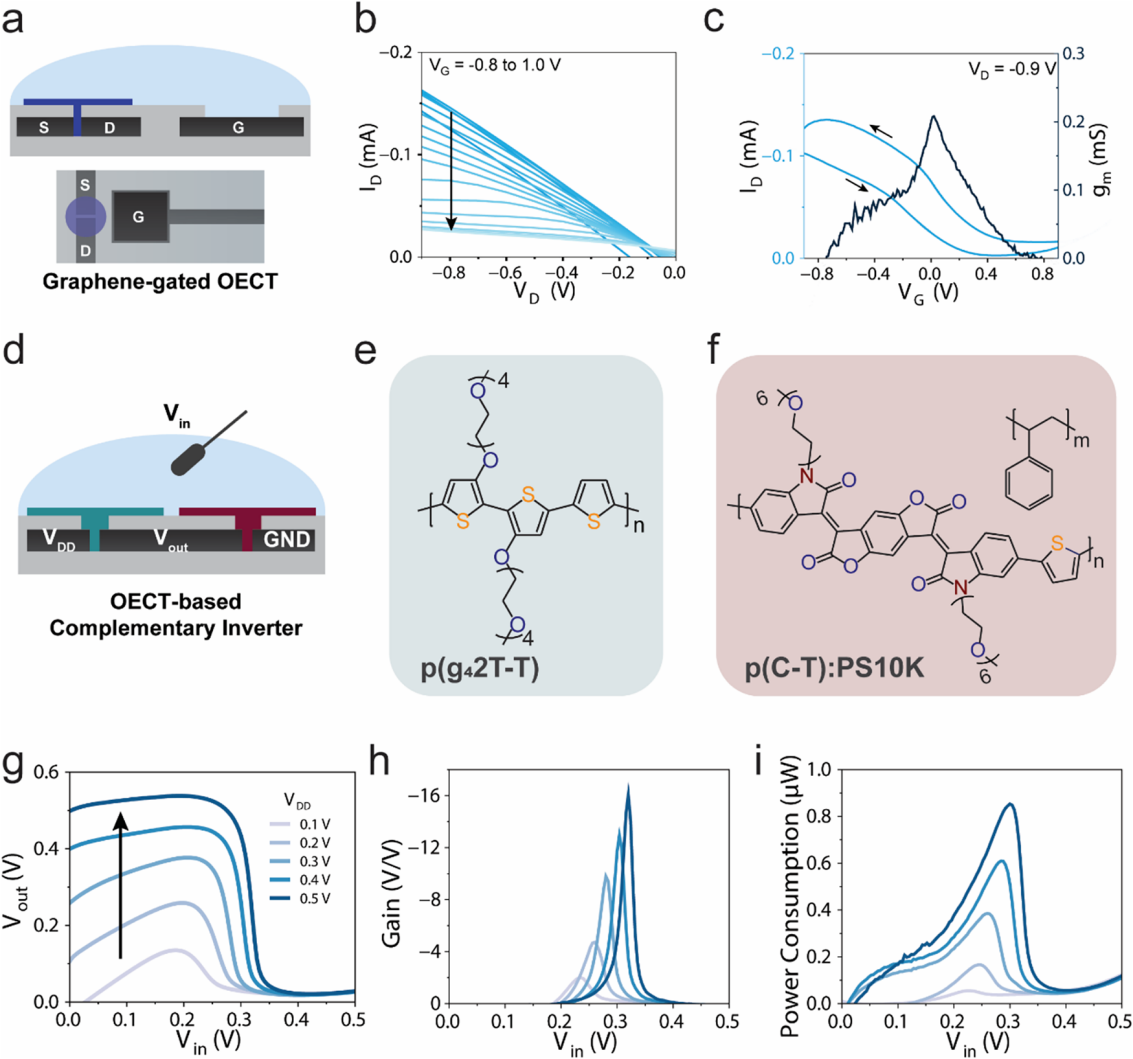
Graphene-gated OECT and OECT-based complementary inverter. (a) Schematic of the graphene-gated *h*OECT with an in-plane graphene gate electrode, represented with both (top) cross-sectional and (bottom) top view. (b) Output characteristics of the graphene-gated *h*OECT, with *V*_D_ ranging from 0 to −V and *V*_G_ from −0.9 to 1 V, with an inset from one of the devices fabricated with this method. (c) Transfer characteristics of the *h*OECT with respect to the gate voltage of planarly patterned graphene electrode (channel *W* = 500 μm, *L = 1* μm, gate = 3000 μm × 3000 μm square). (d) Schematic representation of the OECT-based complementary inverter circuit, where *V*_DD_ is connected to the p-type OECT channel (p­(g_4_2T-T)), with *V*_out_ subsequently leading to the n-type OECT channel (p­(C-T):PS10K 1:6). Both channels are gated with *V*_in_, which serves as the reference electrode. (e, f) Chemical structure of the p-type p­(g_4_2T-T) and n-type p­(C-T):PS10K 1:6. (g) Voltage transfer characteristic, (h) calculated voltage gain, and (i) power consumption characteristics of a complementary inverter based on p­(g_4_2T-T) and p­(C-T):PS10K 1:6 interconnected p/n OECTs.

We measured voltage transfer characteristics of the inverter by sweeping the input voltage from 0 to 0.5 V under incremental supplying voltage (*V*_DD_) steps of 0.1 V, ranging from 0.1 to 0.5 V (Figures 4g and S15). The voltage gain, defined as ∂*V*_out_/∂*V*_ini_, reaches a maximum of 15 V/V at *V*_DD_ = 0.5. Expressed in decibels, the gain corresponds to 20 log_10_(gain) = 23.5 dB, with a switching threshold *V*_M_ of 0.32 V (Figure 4h). These results are in line with previously reported gains for printed complementary OECT inverters.^61–63^

Output currents reveal an asymmetric switching behavior, with bending at low *V*_IN_, attributed to the operation of the p-type transistor, which does not fully turn off at zero gate voltages, unlike the n-type OECT (Figure S15b). Further optimizations, such as tuning the channel dimensions, can be used to improve the match in transient characteristics and further optimize the inverter behavior, but are beyond the scope of this study.^23^

The combination of high gain and low-voltage operation makes OECTs well suited for low-power, biointerfaced sensing in aqueous electrolytes (e.g., NaCl, KCl, PBS) by converting analog (bio)­chemical signals into digital outputs. Also, electrochemical doping and redox behavior of semiconducting polymers amplify small ionic changes, enabling detection of biomolecules in physiological fluids, already demonstrated to work on PEDOT:PSS-OECTs,^64^ as well as pH-sensor OECTs,^65^ wearable,^66^ or implantable platforms.^67^

In addition, we analyzed power consumption by extracting the inverter’s current from its *V*_OUT_/*V*_IN_ characteristics (Figure 4i). Across all *V*_DD_ values, the static power consumption remains below 0.86 μW operation achieved even at the supply voltage of 0.5 V, highlighting the circuit’s suitability for low-power applications. The low-voltage operation and clear inversion demonstrate the platform′s compatibility with a variety of organic mixed ionic/electronic conductors and include more complex transistor configurations to create logic gates with cleanroom-free fabricated flexible devices.

The operation of horizontal, vertical, planar-gated, and inverter OECTs demonstrates that water-based graphene ink and cellulose acetate in acetone enable metal-free devices capable of low-voltage ionic-to-electronic signal transduction on flexible, biobased substrates. Future studies should further investigate how to reduce the resistance of printed electrodes, to mimic metal-like conductivity, and to optimize the OMIEC deposition to enable high-performance OECTs and OECT-based inverter circuits.

### Thermal Degradation and Sustainable Device Lifecycle

Adopting carbon-based materials such as graphene and CA offers a promising route toward more environmentally sustainable OECTs, addressing concerns associated with conventional inorganic components, such as gold and chromium. In contrast to typical insulating polymers used for device passivation, such as Parylene C, which requires chemical vapor deposition, CA can be deposited via a simple room-temperature spin-coating process, reducing energy consumption and fabrication complexity. Similarly, extrusion printing relies on inexpensive equipment and nozzles and enables the printing of water-based inks at room temperature.

To evaluate the environmental footprint of our all-carbon OECTs, we investigated the thermal degradation behavior of devices made of graphene and CA (Figure 5). Traditional OECTs often incorporate metals such as gold, silver, chromium, and titanium, which demand energy-intensive processing due to their high melting points (e.g., gold at 1064 °C, silver at 961 °C, chromium at 1907 °C, and titanium at 1668 °C) and environmentally hazardous recycling methods, such as acid leaching.^68^ In contrast, carbon-based devices can be degraded by thermal processes at significantly lower temperatures.

**Figure 5:**
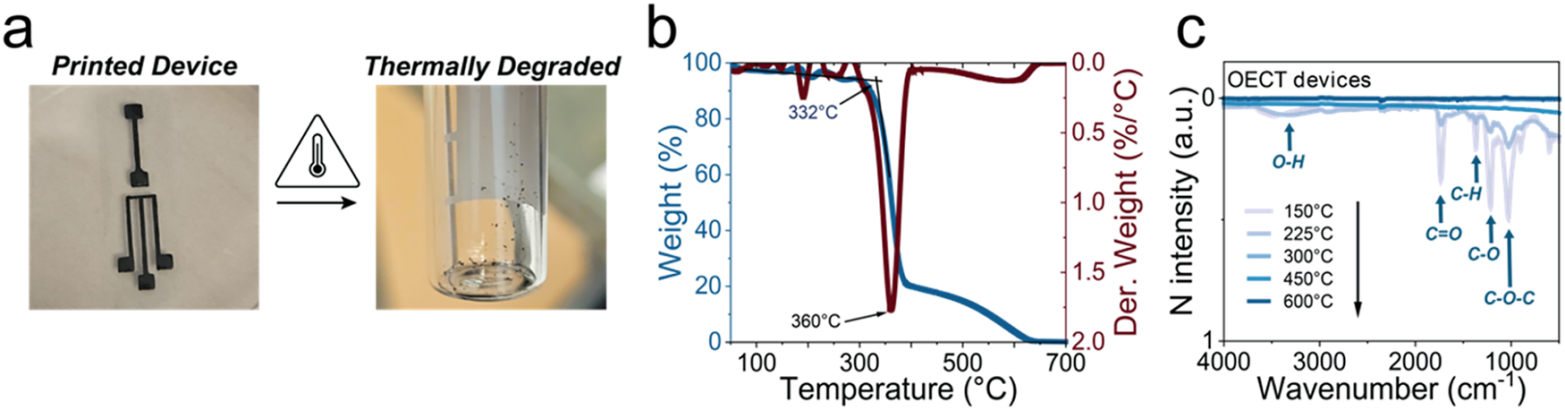
Thermal degradation of all printed *h*-OECTs. (a) Before (left) and after (right) thermal degradation. (b) TGA results and critical temperatures to consider, scanning from room temperature to 700 °C. (c) FTIR spectra for fully fabricated *h*-OECT devices at different temperatures from 150 to 600 °C.

Thermogravimetric analysis (TGA) of our devices, composed of graphene and CA, shows degradation occurring around 360 °C using air in a standard laboratory oven. The thermal decomposition profile up to 700 °C (Figure 5b) reveals an initial decomposition onset at 332 °C, followed by a major degradation peak near 360 °C. This process results in minimal residue (∼0.2% ash by weight), indicating that 80% of the device mass undergoes thermal decomposition at 360 °C.

To further investigate the chemical changes underlying this degradation, we employed Fourier transform infrared (FT-IR) spectroscopy to analyze the thermal breakdown of CA within the *h*OECTs. The FT-IR spectra revealed a significant reduction in characteristic functional groups above 300 °C, including O–H stretching vibrations (3400–3200 cm^–1^), aliphatic C–H stretching (3000–2800 cm^–1^), C=O stretching (1750 cm^–1^), and C–O stretching and C–H bending vibrations (1500–1000 cm^–1^). We observed similar changes in CA controls (Figure S16), confirming that these spectral changes can be attributed to the degradation of ester, hydroxyl, and aliphatic hydrocarbon groups of CA.^22^ The results are consistent with other works, identifying H_2_O, CO_2_, CO, and nonvolatile carbonaceous residues as the main products of cellulose thermal decomposition,^69^ which represent approximately 95% of the total volume per device alongside graphene and conducting polymers.

Given that most disposable devices are ultimately incinerated with municipal solid waste streams,^70^ our results support the feasibility of using low-energy thermal degradation as an end-of-life strategy. This highlights the potential of degradable carbon-based electronics for single-use applications in bioelectronics.

## Conclusions

In this work, we presented a scalable, lithography-free microfabrication strategy for fully organic, self-standing, metal-free, carbon-based, flexible organic electrochemical transistors (OECTs), leveraging a hybrid additive-subtractive approach. By combining spin-coating of cellulose acetate (CA) substrates, extrusion printing of aqueous graphene ink, and femtosecond (fs) laser ablation of CA insulation layers, we achieved micrometer-scale resolution and cleanroom-free device production using degradable, carbon-based materials. We validated the versatility of this approach through the fabrication of multiple OECT architectures, including horizontal-, vertical-, and planar-gated configurations, with channel lengths down to 1 μm and electrode sheet resistances below 10 Ω/sq. This platform supports the integration of functional circuit elements, such as complementary inverters based on p- and n-type of OECTs, and enables the realization of fully patterned, flexible devices with printed graphene gate electrodes. Thermal degradation analysis shows that over 80% of the device mass decomposes below 360 °C, offering a low-energy end-of-life strategy compared to conventional metal-based electronics. Overall, this work bridges sustainable material development with advanced microfabrication, providing a practical route toward the development of high-performance, low-footprint organic bioelectronic devices. By combining high-resolution patterning, environmentally friendly materials, and scalable manufacturing, this approach lays the foundation for next-generation flexible OECT-based systems in wearable, implantable, and disposable sensing applications.

## Supplementary Material











## Notes

Cellulose acetate films characterization; four-point probe sheet resistance and Raman spectra of graphene at different annealing temperatures; 3D optical profilometry methodology and automated feature recognition algorithms; other laser scanning parameter effects on ablation dimensions; fabrication flowcharts for OECT architectures; examples from the downscaling of the ablation in OECT patterning; electrochemical characterization data; FTIR spectra of materials before and after thermal degradation; optical schematic of femtosecond laser workstation (PDF)
Simultaneous ablation of graphene and cellulose acetate using 4× objective (MP4)
Simultaneous ablation with 10× objective at subthreshold power (MP4)
Simultaneous ablation with 20× objective at subthreshold power (MP4)
Selective ablation of cellulose acetate using 4× objective (MP4)
