# Supplementary material for: Microfabricated Organic Electrochemical Transistors Enabled by Printing and Laser Ablation
Source: ACS Appl Mater Interfaces. 2025 Nov 13;17(47):64783–95. doi: 10.1021/acsami.5c16767 (PMC12673519; doi:10.1021/acsami.5c16767)
Supplement: Supplementary file 1 [file am5c16767_si_001.pdf]

Supporting Information

# Microfabricated Organic Electrochemical Transistors Enabled by Printing and Laser Ablation

Alan Eduardo Avila Ramirez[1,‡], Jessika Jessika[1,‡], Yujie Fu[2], Gabriel Gyllensting[3], Marine Batista[3,4], David Hijman[3], Jyoti Shakya[5], Yazhou Wang[6], Wan Yue[7], Renee Kroon[8], Jiantong Li[2], Mahiar Max Hamedi[5], Anna Herland[1,4,*], Erica Zeglio[3,4,*]

[1] Division of Nanobiotechnology, SciLifelab, Department of Protein Science, KTH Royal Institute of Technology, Tomtebodavägen, 23a, 171 65, Solna, Sweden

[2] School of Electrical Engineering and Computer Science, KTH Royal Institute of Technology, Electrum 229, Kista 16440, Sweden

[3] Wallenberg Initiative Materials Science for Sustainability, Department of Chemistry, Stockholm University, 114 18, Stockholm, Sweden

[4] AIMES – Center for the Advancement of Integrated Medical and Engineering Sciences at Karolinska Institutet and KTH Royal Institute of Technology, Stockholm, Sweden

[5] Department of Fibre and Polymer Technology, KTH Royal Institute of Technology, Teknikringen 56, 10044 Stockholm, Sweden.

[6] Organic Bioelectronics Laboratory, Biological and Environmental Science and Engineering (BESE) Division, King Abdullah University of Science and Technology (KAUST), Thuwal, Saudi Arabia

[7] State Key Laboratory of Optoelectronic Materials and Technologies, Key Laboratory for Polymeric Composite and Functional Materials of Ministry of Education, Guangzhou Key Laboratory of Flexible Electronic Materials and Wearable Devices, School of Materials Science and Engineering, Sun Yat-sen University, Guangzhou, 510275 P. R. China

[8] Wallenberg Initiative Materials Science for Sustainability, Laboratory of Organic Electronics, Department of Science and Technology, Linköping University, Norrköping, Sweden.

**Corresponding Author**

* E-mail: erica.zeglio@su.se (E.Z.).

* E-mail: aherland@kth.se (A.H.).

**Supplementary Video 1. Simultaneous ablation of graphene and cellulose acetate using 4× objective.** (mp4 file format).

This video demonstrates real-time femtosecond laser ablation using three passes. As the laser scans over the device, changes in light reflection reveal the formation of the ablated channel. Light angle was adjusted mid-video to enhance visual confirmation of opening depth and uniformity. This method removes both layers efficiently and is used mainly in horizontal OECT fabrication.

**Supplementary Video 2. Simultaneous ablation with 10× objective at sub-threshold power.** (mp4 file format).

The video begins with two passes of sub-threshold power. The second pass partially opens the graphene, and subtle changes in brightness and contrast (adjusted live) help determine whether full ablation is achieved. The third pass shows minimal further expansion, confirming that once the ablation threshold is reached, additional passes have limited effect on lateral dimensions.

**Supplementary Video 3. Simultaneous ablation with 20× objective at sub-threshold power.** (mp4 file format).

Here, more than 10 sequential passes with sub-threshold power are applied using 20× objective. Gradual material removal is observed, leading to complete graphene opening without significant change in lateral opening size. At the end, Z-height is adjusted to confirm complete penetration through the graphene layer.

**Supplementary Video 4. Selective ablation of cellulose acetate using 4× objective.** (mp4 file format).

This video shows progressive ablation of the cellulose acetate encapsulation layer without disturbing the underlying graphene. Initially, oxidation appears as a 'burn mark' (darkening), which starts diminishing on the second pass and becomes brighter on the third, indicating transition to the melting phase. This gradual shift reflects controlled thermal degradation of the cellulose acetate surface, used to expose vertical OECT channels without compromising electrode integrity.

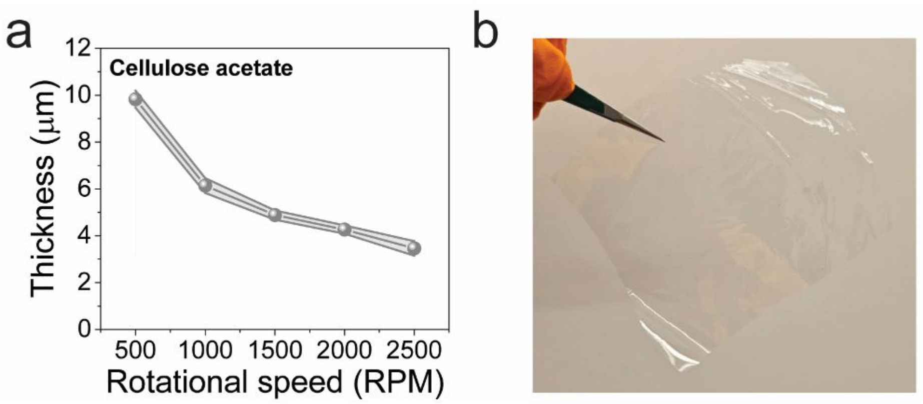


**Figure S1. Cellulose acetate films characterization.** (a) Thickness measurements of the spin-coated cellulose acetate substrate obtained using Dektak profilometer at different revolutions per minute to show the flexibility to change thickness with our developed ink. (b) Photograph of the cellulose acetate film (~5 μm thick) after delamination from the carrier substrate.

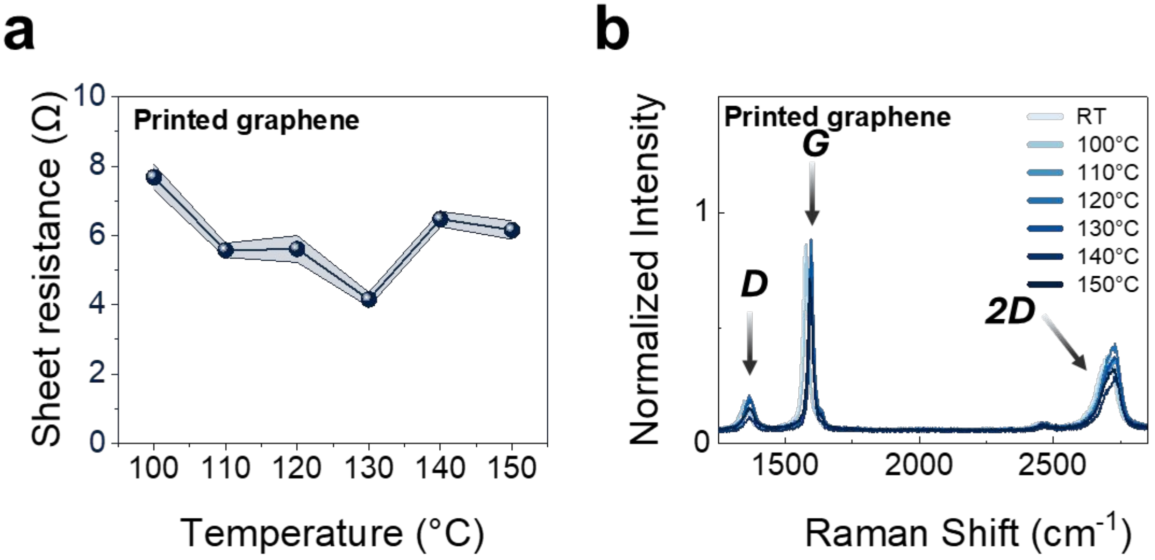


**Figure S2. Printed graphene characterization.** (a) Four-point probe sheet resistance measurements of annealed graphene ink at different temperatures. (b) Raman spectra of printed graphene patterns at different annealing temperatures. The D peak, located at approximately 1350 $cm^{-1}$, is due to the disordered structure of graphene.[1–3] The G peak, located at approximately 1600 $cm^{-1}$, arises from the stretching of the C-C bond of the $sp^2$ hybridized carbons of graphene. The 2D peak, found around 2717 $cm^{-1}$, results from second-order Raman scattering by in-plane transverse optical phonons near the boundary of the Brillouin zone of graphene. The full width at half maximum (FWHM) of the 2D peak for the graphene ink is measured at approximately 80 $cm^{-1}$, confirming the presence of multilayer graphene.

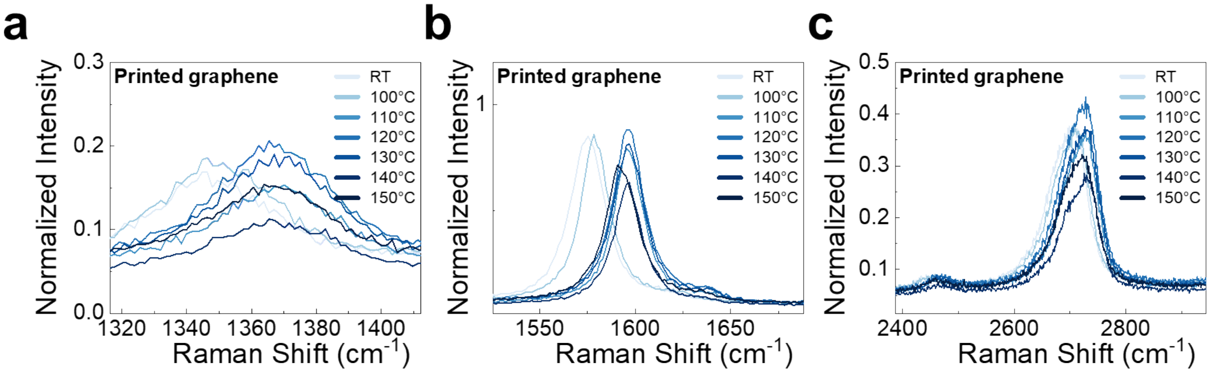


**Figure S3. Raman spectra of printed graphene.** Zoom-ins of the Raman spectra showing the temperature dependance of the position of the (a) D, (b) G, and (c) 2D peaks of printed graphene from 100 °C to 150 °C.

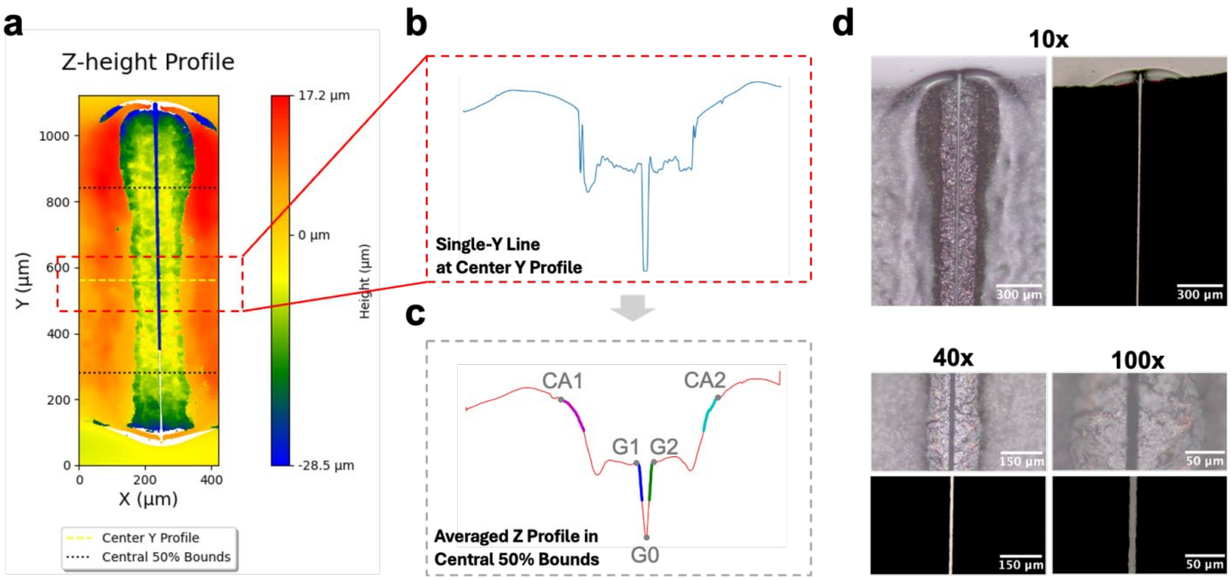


**Figure S4. 3D optical profilometry and automated feature recognition on patterned sample.** (a) Z-height profile colormap of the ablated sample surface, captured using a 3D optical profilometer. The yellow dashed line marks the single-line central Y-profile, while the blue dotted lines indicate the central 50% region for the lateral analysis. (b, c) Extracted Z-height profiles from the central single-line Y-profile and averaged region with key feature points annotated. G1 and G2 represent local maxima on the graphene surface, while G0 is the global minimum corresponding to the deepest point. CA1 and CA2 represent the lateral opening of the cellulose acetate (CA) insulator layer, defined by the first derivative minima (left) and maxima (right). (d) Further verification of successful graphene opening was performed via optical microscopy, showing both the top view of and the transmitted light illumination view of the ablated regions at 10x, 40x, and 100x magnifications to verify graphene exposure and channel clarity.

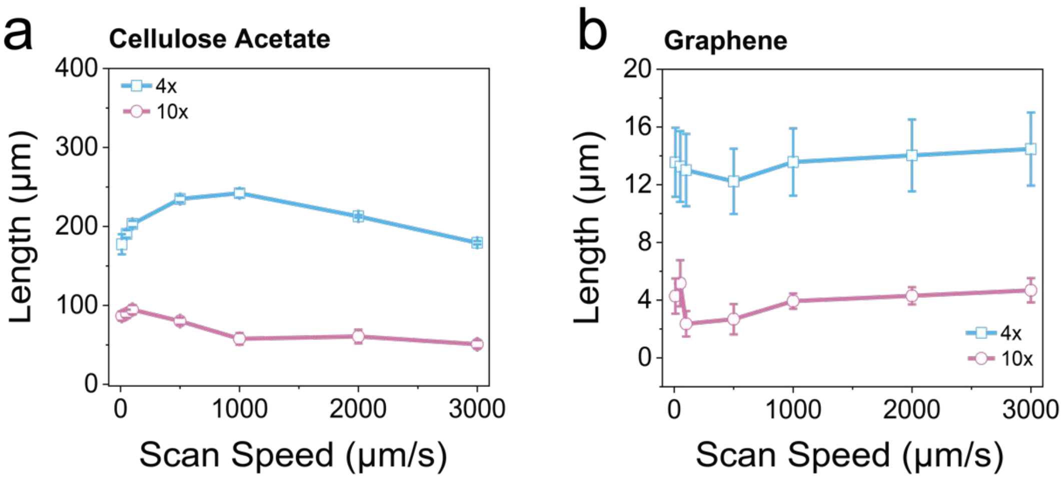


**Figure S5. Influence of speed in laser ablation on lateral opening length.** In (a) graphene, and (b) cellulose acetate.

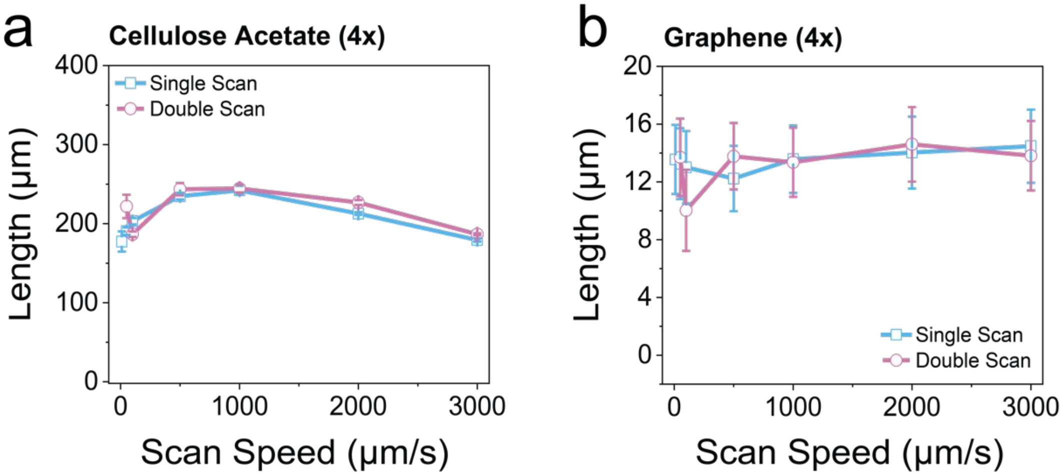


**Figure S6. Influence of pass number on lateral opening length via laser ablation with 4x objective.** In (a) graphene, and (b) cellulose acetate.

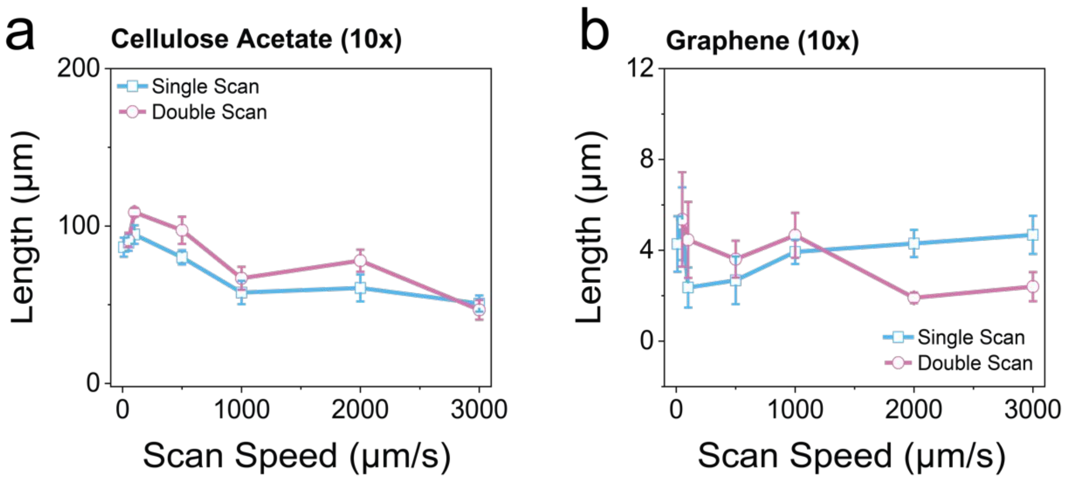


**Figure S7. Influence of pass number on lateral opening length via laser ablation with 10x objective.** In (a) graphene, and (b) cellulose acetate.

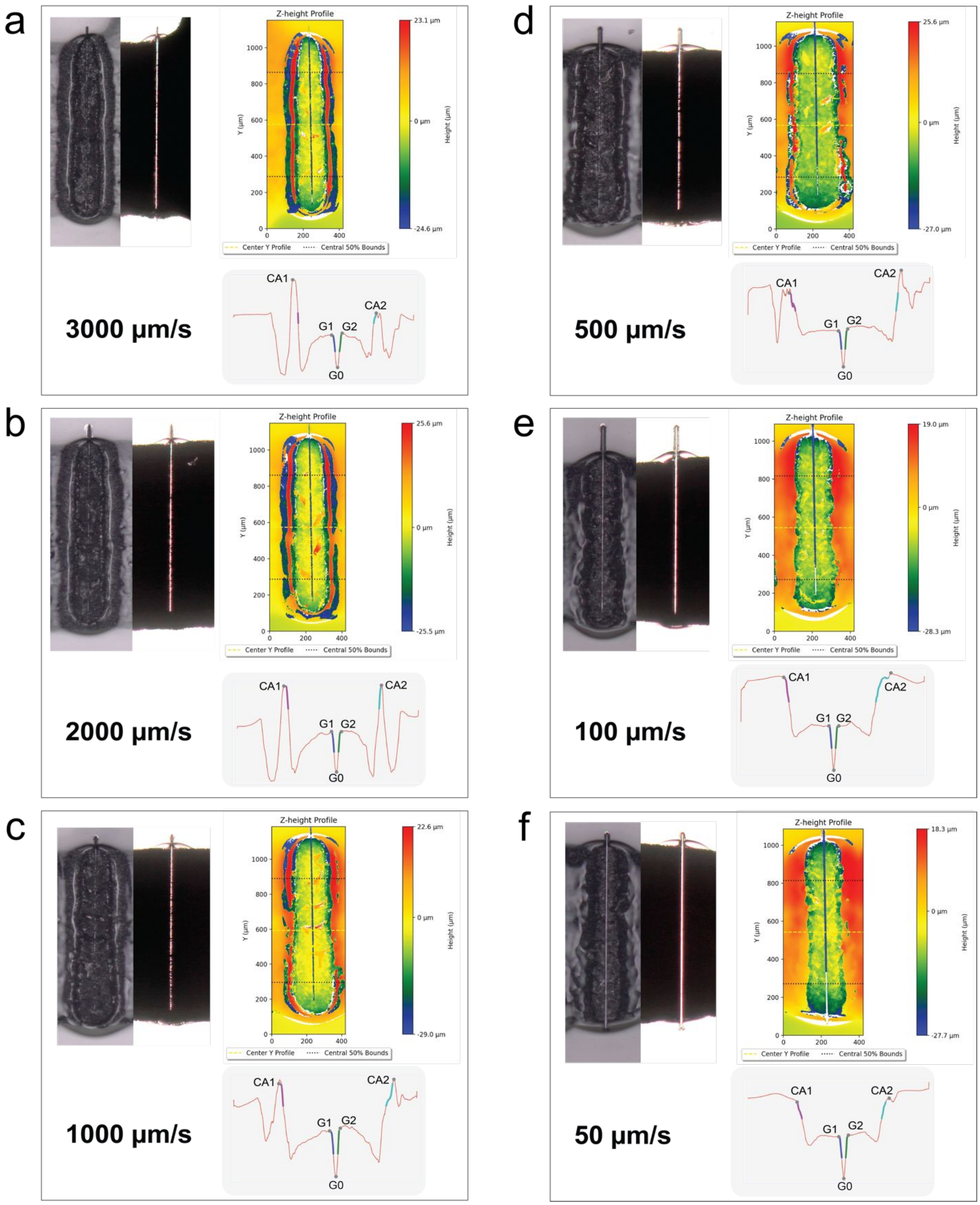


**Figure S8. Effect of scanning speed on graphene and cellulose acetate features with 4x objective.** A series of images and analyses demonstrating the effect of scanning speed on graphene and cellulose acetate lateral openings, performed with 4x objective at 1000 mW and -150 µm Z-height. Each panel shows optical microscopy images of the ablated regions, with reflecting (RL)

and transmitting light (TL) views, for scanning speeds ranging from 3000 µm/s to 50 µm/s. The Z-height profiles obtained using 3D optical profilometer are represented with the colormap indicating height variations and the extracted height profiles from the averaged 50% central Y bounds region, with key feature points for cellulose acetate and graphene annotated as CA1, CA2, G0, G1, and G2.

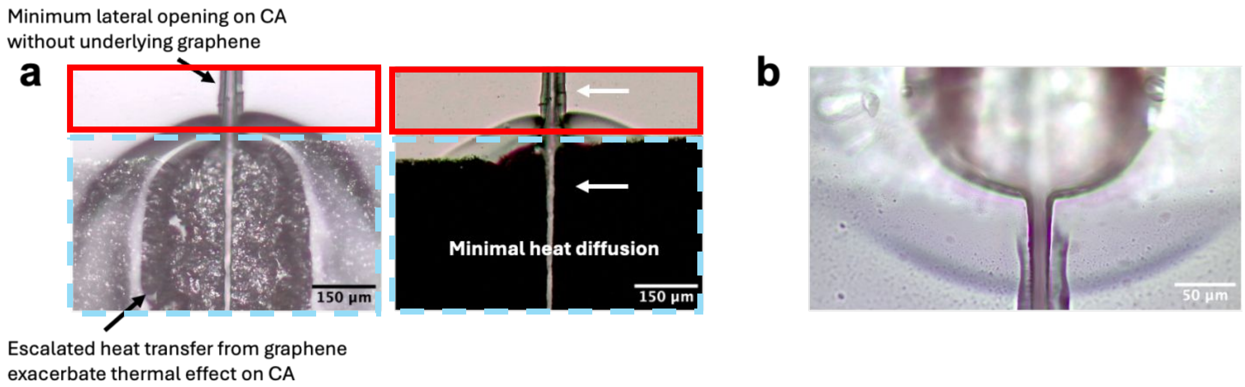


**Figure S9. Graphene-enhanced thermal ablation of cellulose acetate.** (a) Reflecting (left) and transmitting (right) microscope images showing the ablation of the cellulose acetate layer. The presence of underlying graphene significantly enhances thermal diffusion, resulting in a wider lateral opening of cellulose acetate (highlighted in light blue, dashed line). In contrast, regions without graphene underneath (highlighted in red) exhibit limited heat diffusion, leading to a more confined ablation profile. Notably, the lateral opening in these regions closely matches that of the graphene layer, as seen in the right image. (b) Optical microscope image of the cellulose acetate surface after ablation, illustrating how the enhanced thermal conductivity of the underlying graphene increases the efficiency of cellulose acetate ablation through localized heat transfer.

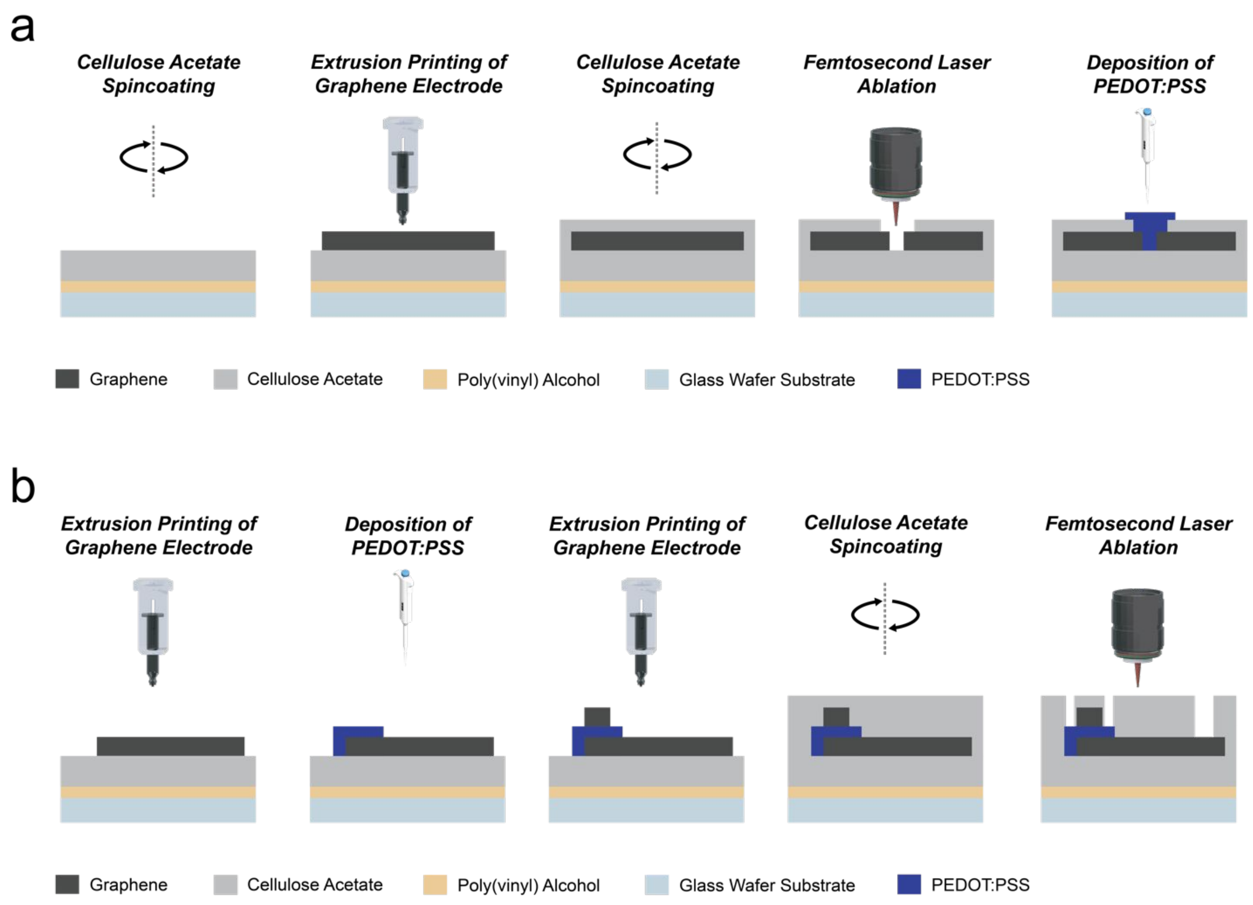


**Figure S10. Fabrication flowcharts for OECT architectures.** (a) Flowchart schematic from the fabrication of hOECT. (b) Flowchart schematic from the fabrication of vOECT.

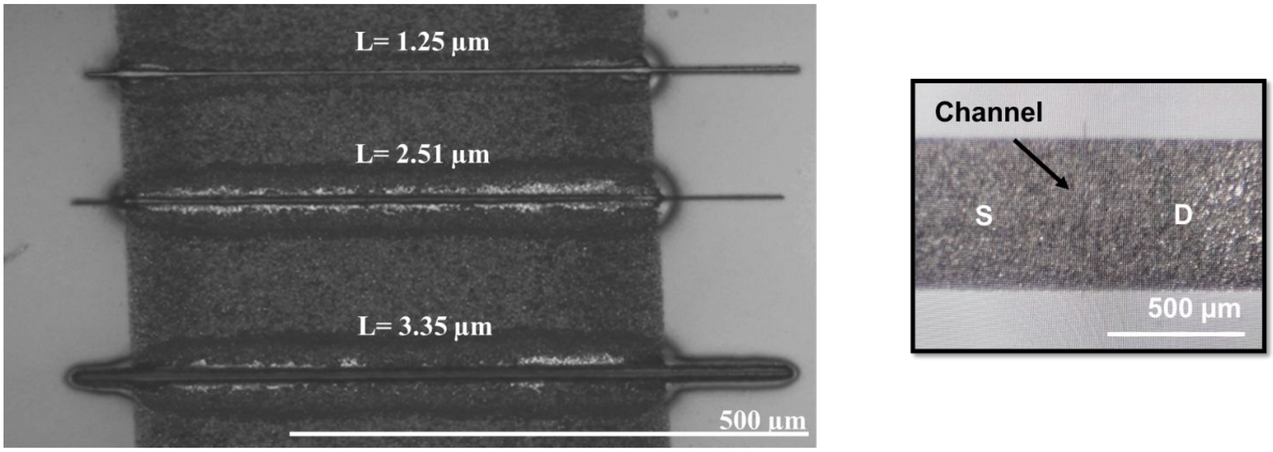


**Figure S11. Examples from the downscaling of the ablation in OECT patterning.** The figure on the left shows examples of the minimum achieved resolution. The example on the right corresponds to an *h*OECT channel prior to the coating of the conducting polymer, shown at a 1 µm scale length opening.

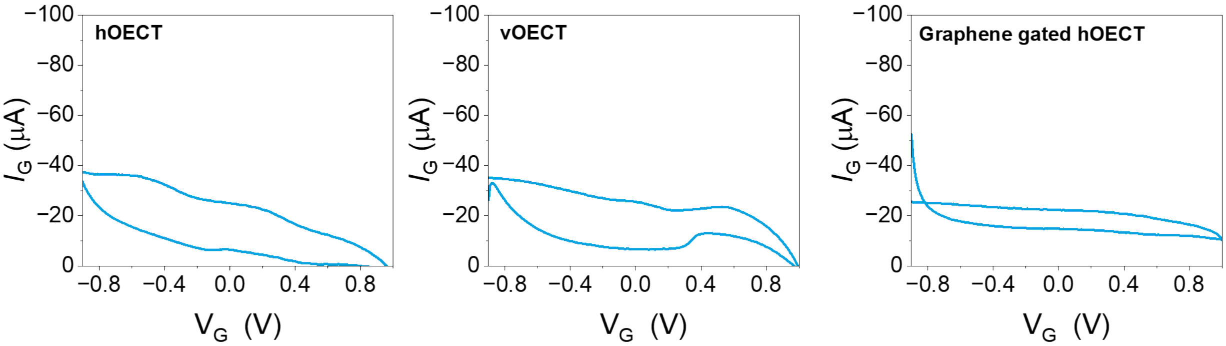


**Figure S12. Gate current from transfer characteristics.** (a) hOECT and (b) vOECT with Ag/AgCl gate electrode; (c) hOECT with gate electrode graphene 3x3 mm.

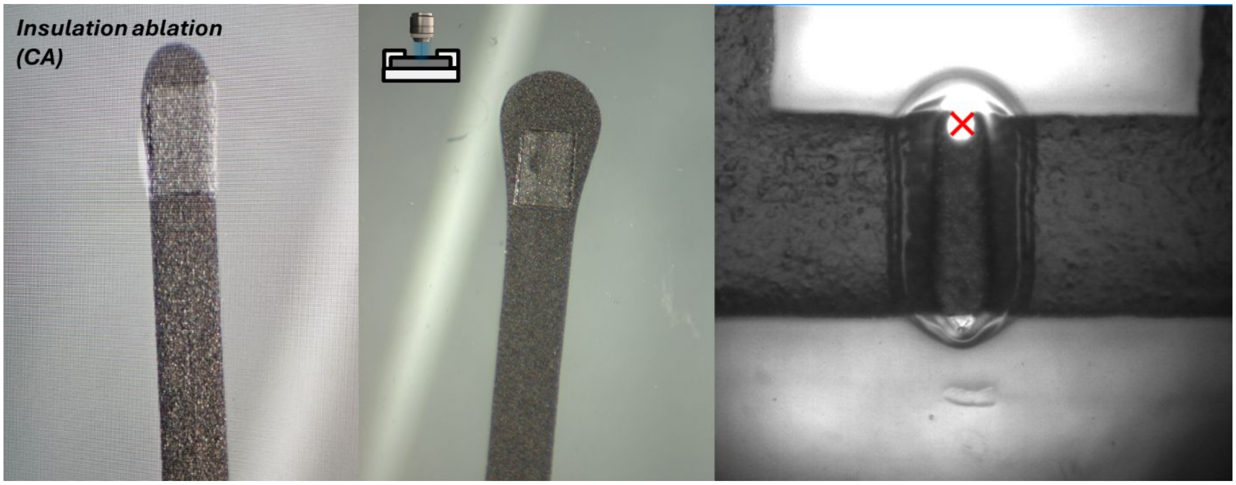


**Figure S13. Selective cellulose acetate ablation.** Representative examples of contact pad openings achieved via selective laser ablation of the cellulose acetate layer, with no observable damage to the underlying printed graphene structures. The third image on the right illustrates the laser in operation, precisely removing the cellulose acetate layer while preserving the integrity of the graphene electrode beneath.

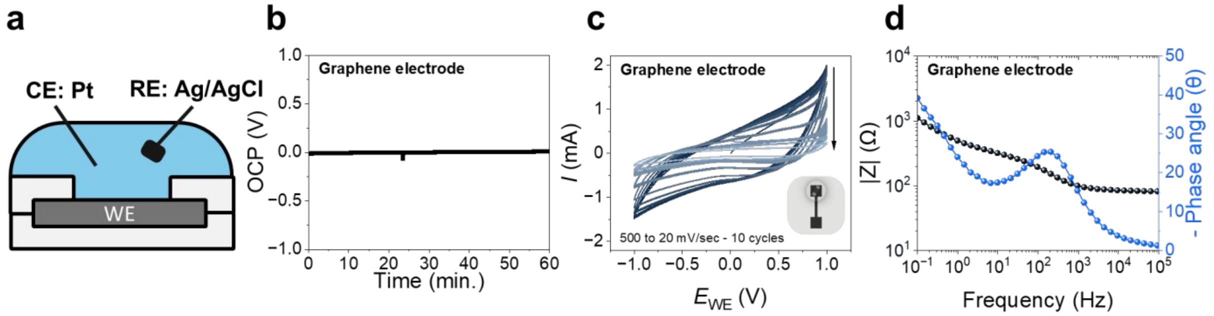


**Figure S14. Electrochemical characterization.** (a) Schematic of a single-graphene electrode after cellulose acetate ablation, used as the working electrode (WE) in a three-electrode setup. (b) Electrochemical stability assessed through cyclic voltammetry at scan rates ranging from 500 mV/s to 20 mV/s, with 10 cycles for each scan rate. (c) Impedance spectroscopy analysis of the electrode, showing low-impedance regimes and capacitive behavior at 0 V.

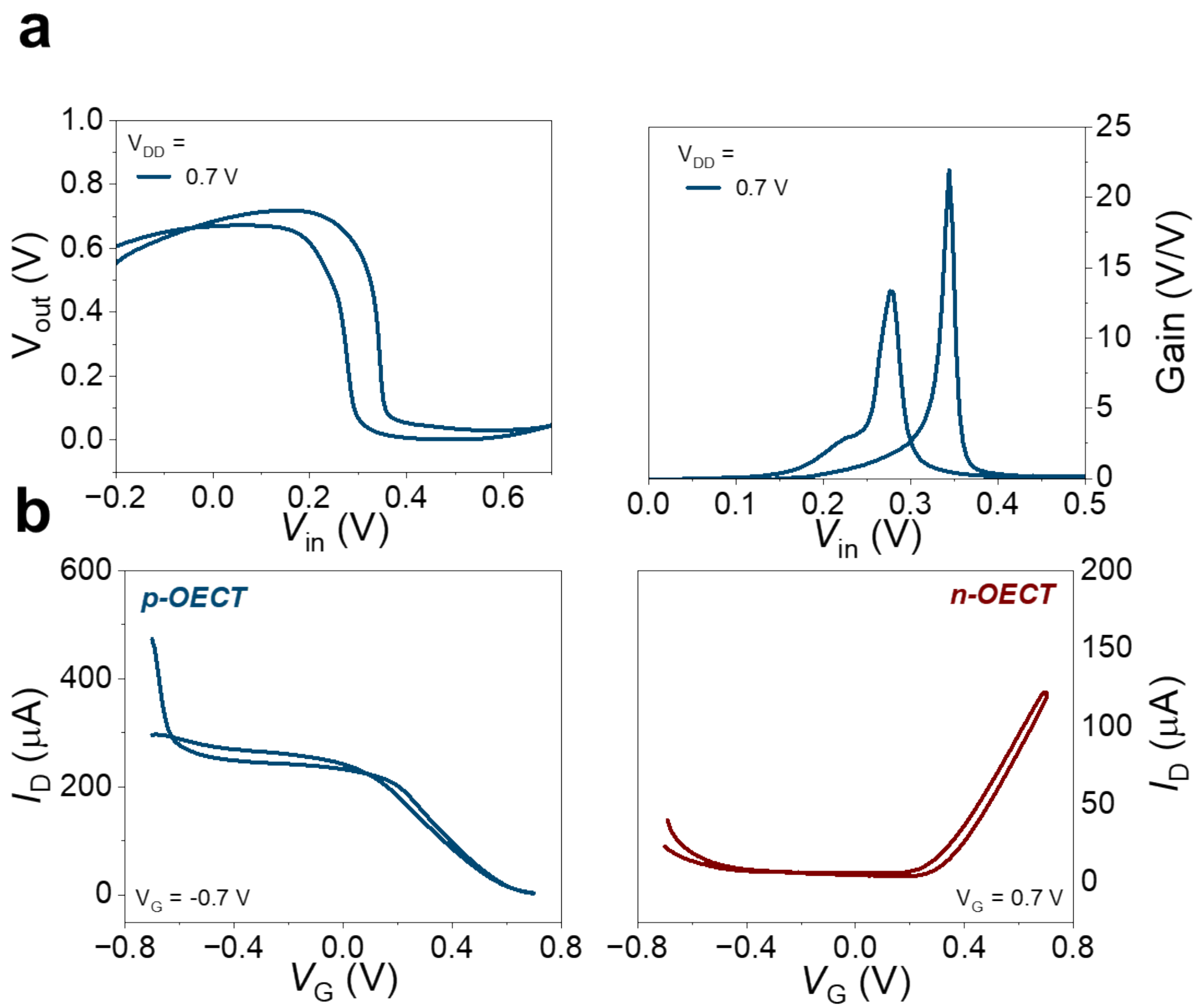


**Figure S15. OECT-based inverter.** (a) Organic complementary inverter operating at a $V_{DD}$ of 0.7 V, above saturation in the initial cycle, resulting in a gain of 22 V/V, comparable to literature values. (b) Transfer characteristics of p(g$_4$2T-T) as the p-type OECT and p(C-T):PS10K 1:6 as the n-type OECT. This represents the highest $V_{DD}$ and $V_{in}$ applied to these devices to avoid degradation at higher potentials.

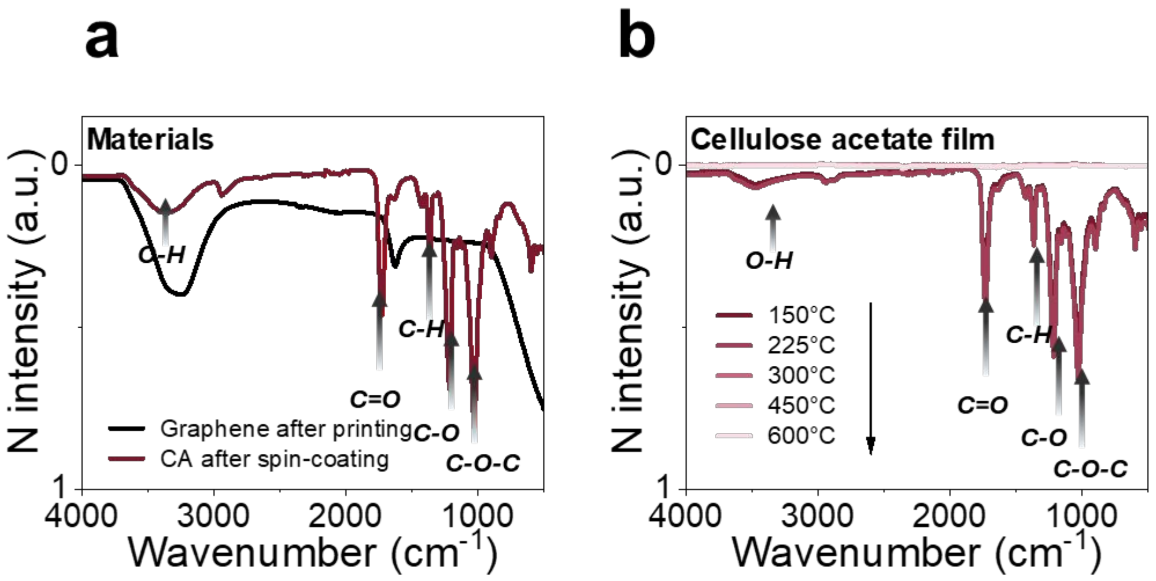


**Figure S16. FTIR spectra of materials before and after thermal degradation.** (a) FTIR scan from raw materials before being degraded. (b) FTIR after degradation at different temperature treatments of the predominant material, cellulose acetate.

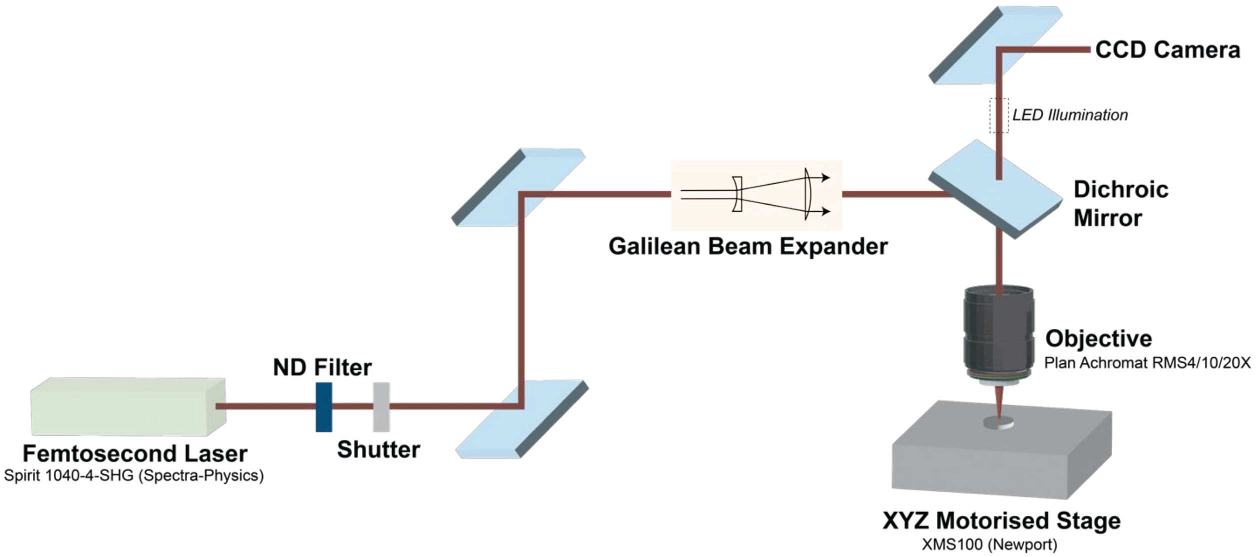


**Figure S17. Schematic diagram of the femtosecond laser workstation for subtractive patterning.** The setup comprises of: (i) femtosecond laser source (Spirit 1040-4-SHG, Spectra-Physics) generating 520 nm pulses at 298 fs duration; (ii) beam conditioning optics including ND filter, shutter, and Galilean beam expander; (iii) a dichroic mirror enabling LED illumination for real-time monitoring through CCD camera and laser delivery through the objective; (iv) interchangeable objectives for focusing the laser beam onto the sample surface: (v) a high-precision XYZ motorized stage (XMS100, Newport) for sample positioning and scanning.

**Supplementary Note 1. Mechanism of femtosecond laser ablation in graphene and cellulose acetate.**

Femtosecond laser ablation enables precise patterning of multilayered materials by leveraging distinct energy transfer mechanism unique to each material. In graphene, ablation occurs via non-linear optical absorption, where the ultrafast laser pulses induce rapid multiphoton ionization and localized plasma formation, enabling sharp and energy-efficient removal of conductive materials.[4] In contrast, cellulose acetate undergoes thermally-driven ablation, in which the absorbed laser energy is converted into heat, inducing localized melting and vaporization. The thermal nature of cellulose acetate ablation results in broader lateral material removal due to heat diffusion. [5]

As shown in **Figure 1** of the main text, two fabrication strategies were employed:

(a) **Simultaneous ablation of both graphene and cellulose acetate**, in which both layers are simultaneously patterned by targeting the graphene surface. This patterning method is suitable for horizontal OECTs requiring both channel separation and direct graphene exposure.

(b) **Selective ablation of cellulose acetate**, achieved by tuning the laser parameters to ablate only the top cellulose acetate layer while preserving the underlying graphene. This method is particularly relevant for fabricating vertical OECTs and planar gates.

**Figure S9** highlights the different ablation mechanisms and illustrates how the presence of graphene underneath enhances the ablation efficiency of cellulose acetate. Microscope images show that, the presence of graphene, laser ablation results in heat-related effects like surface bulging. This is attributed to the strong graphene absorption in the visible and near-IR regions and high thermal conductivity, rapidly heating the film during laser exposure. The heat is transferred upward, increasing the temperature of the overlying cellulose acetate, and thereby enlarging the ablation area while inducing heat-related effects like surface bulging. In the absence of graphene, this pronounced ablation effect disappears, and cellulose acetate exhibits only minimal ablation under identical conditions (**Figure S9a**).

The dependence of cellulose acetate's lateral opening profile on this graphene-induced thermal effects further supports the distinction between graphene's non-linear optical-driven ablation and cellulose acetate's thermal-driven ablation. As shown in **Figure S9b,** post-ablation preview of the cellulose acetate surface depicts the heat-induced bulging and expansion effects.

Selective ablation is achieved by carefully tuning both laser fluence and Z-height (focal position) to ensure the energy delivered exceeds the ablation threshold of cellulose acetate while remaining below that of graphene. Cellulose acetate ablation typically initiates at powers of 300–400 mW (depending on film thickness), whereas graphene ablation begins at around 700–800 mW when using a 4× objective. Operating within this power window allows for clean removal of cellulose acetate without damaging the graphene layer. While Z-height adjustment primarily governs spatial precision, laser power remains the dominant parameter that influences the extent of lateral opening in the cellulose acetate layer.

**Supplementary Note 2. 3D Optical Profilometry Data Processing: Algorithm for automated feature recognition and ablation features dimension measurement from surface profiles.**

To quantify ablation outcomes from varying laser ablation parameters, we employed 3D optical profilometry based on coherence scanning interferometry (CSI) to capture Z-height maps of patterned samples. A custom algorithm was developed to extract key geometric features from these scans by analyzing first- and second-order derivatives of the surface profiles.

The surface height profiles obtained from the raw CSI data was processed to extract key morphological features, with feature detection performed via first and second derivative analysis of the Z-height profile. It begins by loading .datx files, decoding their HDF5 structure, and extracting the Z-height data while converting the pixel data into micrometers scale. The pixel size is retrieved from the metadata to ensure accurate spatial scaling.

The colormap data were divided into a central 50% Y-region to be extracted for averaging to focus on the representative region and minimize edge effects. Feature detection was performed by calculating the first and second derivatives of the Z-height profile. The first derivative was used to manually identify local maxima and minima, while the second derivative was employed to detect inflection points to define key surface features.

Separate feature recognition algorithms were applied for cellulose acetate (CA) and graphene features respectively. For the CA features, CA1 (left) was defined as the local minima of the second derivative from the left, and CA2 (right) was the local maxima of the second derivative from the right. For the graphene features, G1 and G2 were identified as local maxima of the Z-height non-derivative data from the left and right sides of the profile, while G0 representing the global minimum of the Z-height data.

The depth of each feature was calculated as the Z-distances from local extrema between the CA1 or CA2 points to G1 or G2 points, and the G1 or G2 points to G0 points. The lateral opening was determined by averaging the X-distance between the CA1 and CA2 points or between the G1 and G2 points across 50% of the respective depth.

**Figure S4a** presents the Z-height colormap, representing the ablated sample's surface profile. The Z-height profile across the entire X-distance at specific Y-levels (with single Y line as shown in **Figure S4b**) was compiled to produce an averaged Z-height profile over the central 50% of the Y-region, illustrated in **Figure S4c**. On this measurement example, the averaged X-distance between G1 and G2 is 13.55 ± 2.39 μm, followed by CA lateral opening of 177.39 ± 12.69 μm and CA depth of 14.44 μm. During the 3D optical profilometry data analysis, microscope images such as illustrated in **Figure S4d** are used alongside the output of automated results to verify valid openings.

## Supplementary References


(1) Gayathri, S.; Jayabal, P.; Kottaisamy, M.; Ramakrishnan, V. Synthesis of Few Layer Graphene by Direct Exfoliation of Graphite and a Raman Spectroscopic Study. *AIP Advances* **2014**, *4* (2), 027116. https://doi.org/10.1063/1.4866595.

(2) Szirmai, P.; Márkus, B. G.; Chacón-Torres, J. C.; Eckerlein, P.; Edelthalhammer, K.; Englert, J. M.; Mundloch, U.; Hirsch, A.; Hauke, F.; Náfrádi, B.; Forró, L.; Kramberger, C.; Pichler, T.; Simon, F. Characterizing the Maximum Number of Layers in Chemically Exfoliated Graphene. *Sci Rep* **2019**, *9* (1), 19480. https://doi.org/10.1038/s41598-019-55784-6.

(3) Foster, C. W.; Down, M. P.; Zhang, Y.; Ji, X.; Rowley-Neale, S. J.; Smith, G. C.; Kelly, P. J.; Banks, C. E. 3D Printed Graphene Based Energy Storage Devices. *Sci Rep* **2017**, *7* (1), 42233. https://doi.org/10.1038/srep42233.

(4) Bobrinetskiy, I. I.; Emelianov, A. V.; Lin, C.-L.; Otero, N.; Romero, P. M. Ultrafast Laser Patterning of Graphene. In *Nanotechnology VIII*; SPIE, 2017; Vol. 10248, pp 108–113. https://doi.org/10.1117/12.2265430.

(5) Harilal, S. S.; Freeman, J. R.; Diwakar, P. K.; Hassanein, A. Femtosecond Laser Ablation: Fundamentals and Applications. In *Laser-Induced Breakdown Spectroscopy: Theory and Applications*; Musazzi, S., Perini, U., Eds.; Springer: Berlin, Heidelberg, 2014; pp 143–166. https://doi.org/10.1007/978-3-642-45085-3_6.
